# Extrinsic Macrophages Protect While Tendon Progenitors Degrade: Insights from a Tissue Engineered Model of Tendon Compartmental Crosstalk

**DOI:** 10.1002/adhm.202100741

**Published:** 2021-09-08

**Authors:** Tino Stauber, Maja Wolleb, Anja Duss, Patrick K. Jaeger, Irina Heggli, Amro A. Hussien, Ulrich Blache, Jess G. Snedeker

**Affiliations:** ^1^ Department of Orthopedics Balgrist University Hospital University of Zurich Lengghalde 5 Zurich 8008 Switzerland; ^2^ Institute for Biomechanics ETH Zurich Zurich 8093 Switzerland; ^3^ Center of Experimental Rheumatology Department of Rheumatology University Hospital, University of Zurich Lengghalde 5 Zurich 8008 Switzerland; ^4^ Fraunhofer Institute for Cell Therapy and Immunology 04103 Leipzig Germany

**Keywords:** crosstalk, ex vivo tissue, macrophages, progenitors, tendons

## Abstract

Tendons are among the most mechanically stressed tissues of the body, with a functional core of type‐I collagen fibers maintained by embedded stromal fibroblasts known as tenocytes. The intrinsic load‐bearing core compartment of tendon is surrounded, nourished, and repaired by the extrinsic peritendon, a synovial‐like tissue compartment with access to tendon stem/progenitor cells as well as blood monocytes. In vitro tendon model systems generally lack this important feature of tissue compartmentalization, while in vivo models are cumbersome when isolating multicellular mechanisms. To bridge this gap, an improved in vitro model of explanted tendon core stromal tissue (mouse tail tendon fascicles) surrounded by cell‐laden collagen hydrogels that mimic extrinsic tissue compartments is suggested. Using this model, CD146^+^ tendon stem/progenitor cell and CD45^+^F4/80^+^ bone‐marrow derived macrophage activity within a tendon injury‐like niche are recapitulated. It is found that extrinsic stromal progenitors recruit to the damaged core, contribute to an overall increase in catabolic ECM gene expression, and accelerate the decrease in mechanical properties. Conversely, it is found that extrinsic bone‐marrow derived macrophages in these conditions adopt a proresolution phenotype that mitigates rapid tissue breakdown by outwardly migrated tenocytes and F4/80^+^ “tenophages” from the intrinsic tissue core.

## Graphical abstract

Tendons are among the most mechanically stressed and damaged tissues of the body. The improved in vitro model of explanted tendon core stromal tissue surrounded by cell‐laden, biofidelic collagen hydrogels re‐establishes tendons in in vivo compartmentalization. In a stressed niche, it captures key features and characteristics of tendon pathology, providing a powerful tool for study of tendon disease, injury, and repair.

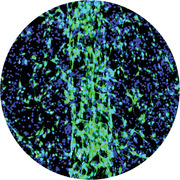

## 1 Introduction

Tendons tissues bear extreme mechanical stresses in their function of transferring muscle forces to bone.^[^ ^1^, ^2^ ^]^ Tendon function relies on its structure of aligned and crosslinked type‐1 collagen fibers that are maintained by a stromal, mostly fibroblastic cell population (tenocytes).^[^ ^3^ ^]^ Stromal cells and collagen fibers together are bundled into a fascicle, the fundamental functional tendon unit.^[^ ^2^, ^4^, ^5^ ^]^ The fascicles of a tendon form the tendon core, a mostly avascular tissue. This load‐bearing core tissue is surrounded and nourished by a synovial‐like tissue layer, which makes up the extrinsic tendon compartment.^[^ ^6^, ^7^, ^8^ ^]^ We have recently studied tendon tissue response to core matrix damage and found a very limited intrinsic capacity for self‐repair.^[^ ^9^ ^]^ It therefore seems plausible that the damaged tendon core relies heavily on recruited reparative cells that either reside in the surrounding extrinsic compartment or are delivered there through the local vasculature.^[^ ^7^, ^10^, ^11^, ^12^, ^13^, ^14^ ^]^ In fact, the looser structure of the extrinsic compartment permits more matrix‐breaching capillaries that can sustain a substantially higher degree of tissue turnover than the core.^[^ ^2^, ^15^, ^16^, ^17^, ^18^, ^19^ ^]^ Mediated by resident tendon stem/progenitor cells and circulating immune cells like macrophages,^[^ ^20^ ^]^ this turnover maintains tissue health and homeostasis by preventing (core) damage accumulation.^[^ ^14^, ^21^, ^22^, ^23^, ^24^ ^]^ Ultimately, the tendon core and the extrinsic compartment fulfill separate but complementary functions to sustainably enable tendon performance.^[^ ^4^ ^]^

However, excessive biochemical and mechanobiological stress can drive dysmetabolism and eventual tendon injury. Tendon disease features a disturbed tendon compartmentalization, with altered spatial chemical gradients of oxygen, nutrients, and growth factors.^[^ ^25^, ^26^ ^]^ This is associated with, and perhaps driven by, accumulated damage and mechanical disruption of collagen fibers (cellular scale “matrix unloading”), with dysregulation of key biophysical cues that regulate the various subcompartmental tendon niches across their interfaces.^[^ ^27^, ^28^ ^]^

The present work pursues the thesis that a tendon core within a biochemically/mechanobiologically stressed niche will activate and recruit resident progenitor and circulating immune cells from the extrinsic compartment in an attempt to remove damaged, unloaded tissue and restore mechanobiological homeostasis.^[^ ^24^, ^29^, ^30^, ^31^, ^32^ ^]^ Failure of the tissue to repair such damage is thought to underlie tendinopathy, a painful syndrome characterized by persisting inflammation, matrix disorganization, and compromised organ compartmentalization/stability that can eventually result in whole tendon rupture.^[^ ^4^, ^26^, ^33^ ^]^ Mechanistic understanding and the development of effective tendinopathy treatments have so far been hindered by shortcomings in available experimental models. Until now, in vivo models of tendinopathy have been limited in their ability to capture essential features of the disease, as well as being time‐consuming, expensive, and difficult to upscale. In vivo models (e.g., genetic mouse models) also present technical and logistical challenges to mechanistically probing key multicellular and multitissue interactions. To overcome some of these limitations, in vitro models, including tissue‐engineered 3D culture systems, have been developed and optimized in the last decades. However, these models have also failed to adequately reproduce critical aspects of in vivo musculoskeletal (mechano‐) biology and pathology. We suspect that this may be due, at least in part, to a lack of the compartmental tissue–tissue interfaces that govern essential spatiotemporal biochemical gradients regulated within a mechanically active microenvironment.^[^ ^8^, ^34^, ^35^ ^]^

We have therefore bioengineered a hybrid tissue model consisting of tendon fascicle explants (mimicking the intrinsic tendon core) surrounded by cell‐laden collagen hydrogels (mimicking an extrinsic compartment). This novel tissue model approach has several advantages: a) the native pericellular matrix of core‐resident cell populations is preserved in the fascicle explant; b) the crosstalk of the tendon core (explant) with extrinsic subcompartments (stromal tendon stem/progenitor cells and bone‐marrow derived macrophages) across a biomimetic cross‐compartmental tissue–tissue interface can be studied in a controllably defined biochemical niche; c) the emergent behavior of the engineered tissue model can be functionally characterized by mechanical testing.^[^ ^36^ ^]^ In the present work, we exploit a versatile “tenostruct” system to recapitulate the multicellular events following a disturbed tissue homeostasis/microenvironment, using it to unwind cellular crosstalk and emergent construct behavior secondary to mimicked tissue injury.

## 2 Results

### 2.1 Tenostruct Fabrication and Characterization

**Figure 1:**
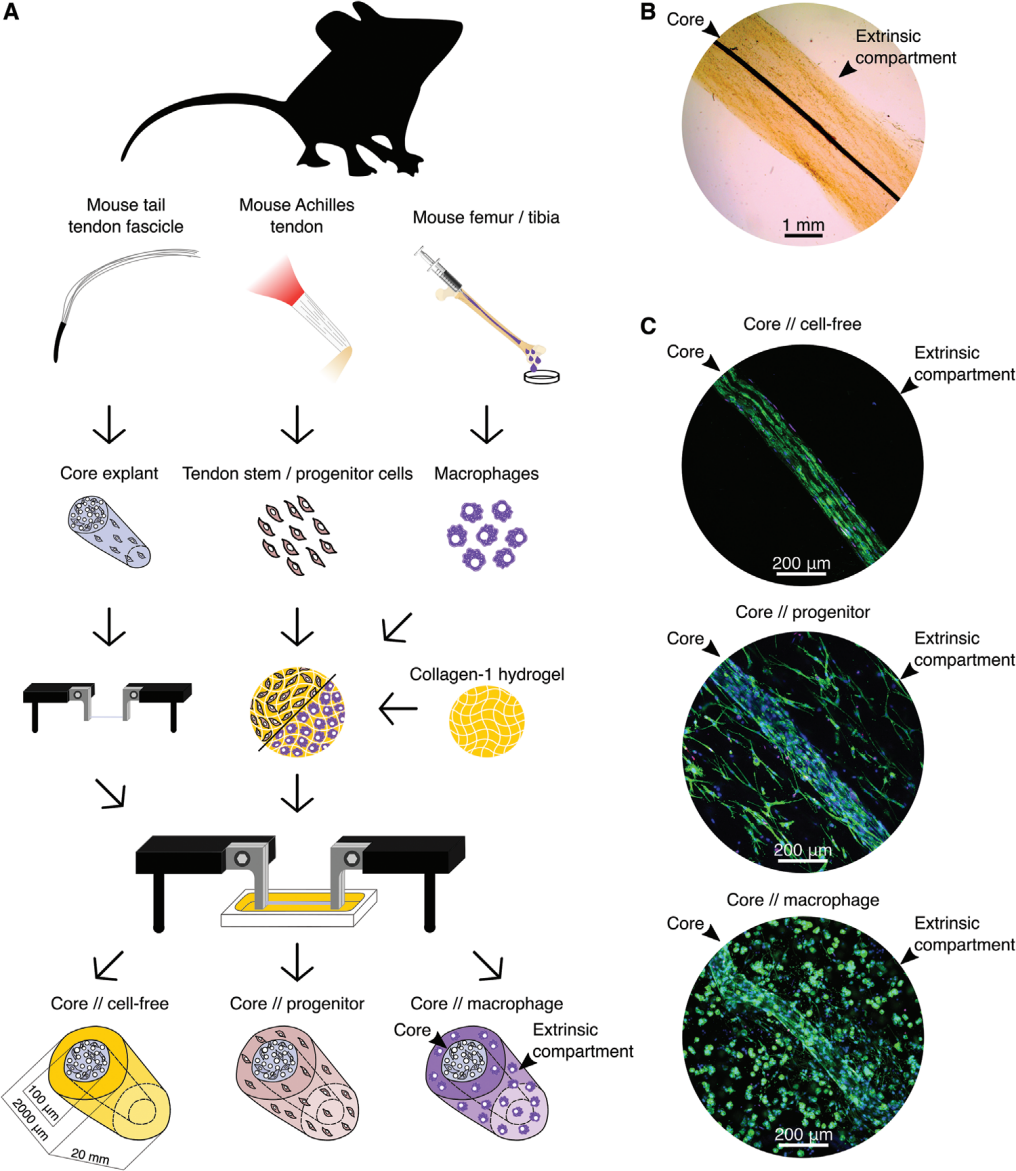
Engineering tenostruct tissue models for the investigation of tendon compartment crosstalk. A) Tendon fascicles as a model of the tendon core tissue were isolated from mouse tails. CD146^+^ tendon stem/progenitor cells were extracted from mouse Achilles tendons. Monocytes were isolated from mouse bone‐marrow and differentiated into naïve macrophages. For tenostruct fabrication, progenitors or macrophages were embedded in a collagen‐1 hydrogel and cast in a silicone‐mold surrounding the tendon core explant, which was anchored in an elongated position between metal clamps. The resulting tenostructs consist of a tendon core surrounded by an extrinsic cell‐free/progenitor/macrophage compartment. B) Representative bright field image of a (core//progenitor) tenostruct. C) Representative immunofluorescence images of a core//cell‐free, a core//progenitor, and a core//macrophage tenostruct with stained cell nuclei (DAPI, blue) and F‐actin cytoskeleton (Phalloidin, green) at day 3.

A tenostruct tissue model was established by combining tendon core explants with hydrogel encapsulated CD146^+^ tendon stem/progenitor cells or bone‐marrow derived macrophages (**Figure** **1**). To erect the stromal core of the constructs, tendon fascicles were carefully extracted from mouse tails by pulling and then mounted to custom‐made metal clamps (Figure 1A, left side; Figure S3, Supporting Information). To add the engineered extrinsic progenitor compartment, we isolated CD146^+^ cells from mouse Achilles tendons by plastic adherence growth for 2–4 passages (Figure 1A, mid). The progenitor‐like phenotype of these cells was verified via the presence of the CD146 surface marker, which only 3.51% of cells in freshly digested tendon core explants and 14.7% of cells in freshly digested Achilles tendons express, but 78.7% and 93.5% of mouse Achilles tendon‐derived cells at passage 2 and 4, respectively (Figure S1, Supporting Information). To engineer the extrinsic macrophage model variant, monocytes were isolated from mouse femur bone‐marrow and differentiated to naïve macrophages (Figure 1A, right side). We verified the expression of macrophage surface markers (CD45 and F4/80) using flow cytometry and their specialization potential through cell morphology and marker expression for M1‐like (*CD86* and *Tnf‐α*) and M2‐like phenotypes (*Mcr1*, *Chi3l3*) upon chemical stimulation with IFN‐*γ* + LPS and IL‐4, respectively (Figure S2, Supporting Information). Then, either a progenitor or macrophage‐laden hydrogel (or a cell‐free control hydrogel) was cast around the clamped tendon core explant using a silicone‐mold (Figure 1A). In summary, three variants of the tenostruct model were created consisting of an explanted tendon fascicle core surrounded by a cell‐free hydrogel (Figure 1A–C, core//cell‐free) or one containing either progenitors (Figure 1A–C, core//progenitor) or bone‐marrow derived macrophages (Figure 1A–C, core//macrophage). In addition, progenitor or macrophage laden control hydrogels were cast in same‐sized silicone molds to serve as single‐culture controls (Figure S3A,D, Supporting Information).

### 2.2 Viability in All Tenostruct Subcompartments Is High, with Extrinsic Progenitors and Outwardly Migrated Cells Elongating along the Tensional Axis of the Core

**Figure 2:**
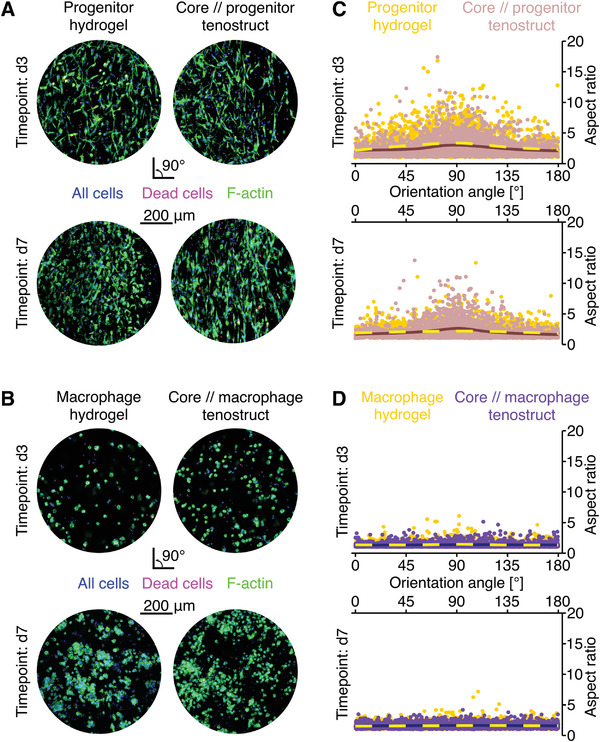
Viability and cell orientation of extrinsic compartment cell populations in hydrogels or tenostructs. Representative immunofluorescence images of A) progenitors and B) macrophages initially seeded in collagen hydrogels and tenostructs with stained nuclei (DAPI, blue), dead cells (EthD‐1, red), and F‐actin cytoskeleton (Phalloidin, green). The morphology plots show cellular aspect ratio and orientation angle (relative to the transversal plane) of C) progenitors and D) macrophages in a hydrogel/tenostruct. Each dot represents an individual cell and the trendline depicts the conditional mean calculated through locally estimated scatterplot smoothing (LOESS) (3000 randomly selected cells from *N* = 3 samples).

We assessed the viability and cell orientation of tenostruct‐embedded cells by live/dead and actin staining, comparing them to cells that were encapsulated in single‐culture control hydrogels. Both progenitor (**Figure** **2**A) and macrophage (Figure 2B) viability was high in control hydrogels as well as in the tenostructs; characteristically few dead cells were detected in either format at both 3 and 7 days.

Mechanical anisotropy (i.e., highly elongated and aligned cells and matrix) is a defining feature of native stromal tendon tissue,^[^ ^37^ ^]^ and known to drive progenitor tenogenicity.^[^ ^38^, ^39^, ^40^ ^]^ Therefore, we investigated the orientation of cells in the extrinsic compartment of core//progenitor tenostructs and control progenitor hydrogels by measuring cytoskeletal angle and aspect ratio (Figure 2A–C). Already on day 3, cells in the tenostructs were elongated and aligned longitudinally (with the embedded core explant) with increasing longitudinal alignment at day 7. By contrast, cells in progenitor hydrogels were also elongated on day 3, but less longitudinally aligned (Figure 2A–C). By day 7, cells in the progenitor hydrogels had assumed a more rounded, nonaligned morphology. Meanwhile, the rounded macrophages in core//macrophage tenostructs or macrophage hydrogels did not elongate with the embedded fascicle at any assessed timepoint (Figure 2B–D). These changes in cell morphology correlated with changes in macroscopic tenostruct/hydrogel appearance over time. After 7 days in culture, the progenitor control hydrogel was contracted longitudinally and radially by the resident cells, while cell‐free and macrophage control hydrogels were not (Figure S3A, Supporting Information). By contrast, only radial contraction was observed in the tenostructs (Figure S3A,B, Supporting Information). Here, the changes were more pronounced in core//progenitor and core//macrophage compared to core//cell‐free tenostructs (Figure S3B, Supporting Information).

### 2.3 Extrinsic Progenitors Accelerate Degradation of Underloaded Core Tissue While Extrinsic Macrophages Protect Tendon Mechanical Properties

**Figure 3:**
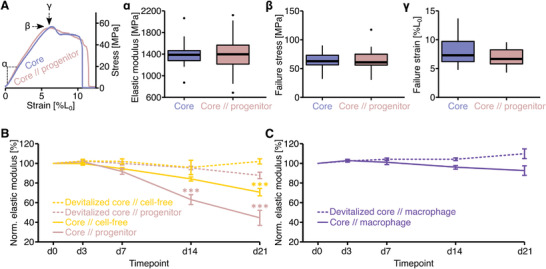
Mechanical properties of engineered tenostructs. A) Representative stress–strain curve of a (core//progenitor) tenostruct in comparison to a tendon core explant at day 0. The mechanical hallmarks are indicated with the number of the respective subfigure (*α*–*γ*). Boxplots are comparing the mechanical hallmarks of (core//progenitor) tenostructs to matched tendon core explants: linear elastic modulus (*α*), failure stress (*β*), and failure strain (*γ*). *N* = 15. The upper and lower hinges correspond to the first and third quartile (25th and 75th percentile) and the middle one to the median. Whiskers extend from the upper/lower hinge to the largest/smallest value no further than 1.5 times the interquartile range. Data beyond the whiskers are depicted as dots. We did not detect significant differences. B) Linear elastic moduli of core//cell‐free tenostructs, core//progenitor tenostructs, and core//cell‐free / core//progenitor tenostructs with a devitalized core explant over a time course of 21 days. C) Linear elastic moduli of core//macrophage tenostructs and core//macrophage tenostructs with a devitalized core explant over a time course of 21 days. Datapoints in both B and C are normalized to the initial linear elastic modulus (d0) of the sample. *N* = 8. The data are displayed as mean (± sem). Results of the statistical analysis of timepoints relative to d0 are indicated as follows: ***p < 0.001. The applied statistical tests were ANOVA followed by Tukey Post‐Hoc.

First, we tested the mechanical properties of engineered tenostructs with an extrinsic progenitor compartment immediately after tenostruct fabrication. To do so, we divided a tendon core explant into two subunits, embedded one in a progenitor‐laden collagen hydrogel and measured the mechanical properties of both subunits after a 1 h gelation time. We did not detect differences in neither the whole stress–strain curve (**Figure** **3**A) nor in well‐established mechanical hallmarks (linear elastic modulus, failure strain, and failure stress (Figure 3A, *α*–*γ*)) of tenostructs compared to unmodified core explants. Briefly, the median (IQR) linear elastic moduli of tenostructs/unmodified core explants were 1397 (352) MPa / 1387 (186) MPa, the median (IQR) failure stresses 61 (19) MPa / 63 (17) MPa, and the median (IQR) failure strains 6.7 (2.5)% / 7.3 (3.5)%. In consequence, the mechanical properties of our tenostructs are mainly determined by the core explants, which is comparable to their load‐bearing function in vivo.

We then exposed clamped tenostructs to conditions resembling a stressed tendon injury niche (elevated tissue oxygenation, temperature, glucose‐levels, growth factor concentration through serum supplementation, and reduced matrix loading through explanting).^[^ ^27^ ^]^ As a longitudinal mechanical readout, we measured the linear elastic modulus of the tendon core explants over a time course of 21 days.

The mechanical integrity of core//cell‐free tenostructs remained comparable to that of matched devitalized tendon core explants in a cell‐free hydrogel until at least d7 (Figure 3B). However, we detected a significant drop in the linear elastic modulus of these core//cell‐free tenostructs starting between day 7 and day 21 (yellow, continuous line); this loss of mechanical integrity was actively mediated by the core since it did not occur with a devitalized core explant (yellow, dotted line). While the extrinsic compartment of core//progenitor tenostructs led to a more rapid core degeneration compared to the cell‐free controls (Figure 3B, freshly isolated: beige, continuous line/devitalized: beige, dotted line), the extrinsic compartment of core//macrophage tenostructs prevented the onset of core tissue breakdown and loss of mechanical properties (Figure 3C). Interestingly, core explants cocultured with a physically separated progenitor‐laden hydrogel (Figure S4, core/–/progenitor, Supporting Information) degraded almost identically to core explants cultured with a cell‐free hydrogel (Figure S4, core/–/cell free, Supporting Information) or a macrophage‐laden hydrogel (Figure S4, core/–/macrophage, Supporting Information).

### 2.4 Scx‐Lineage Progenitors Recruit to Damaged Core, Core‐Resident Tenocytes Migrate Out

**Figure 4:**
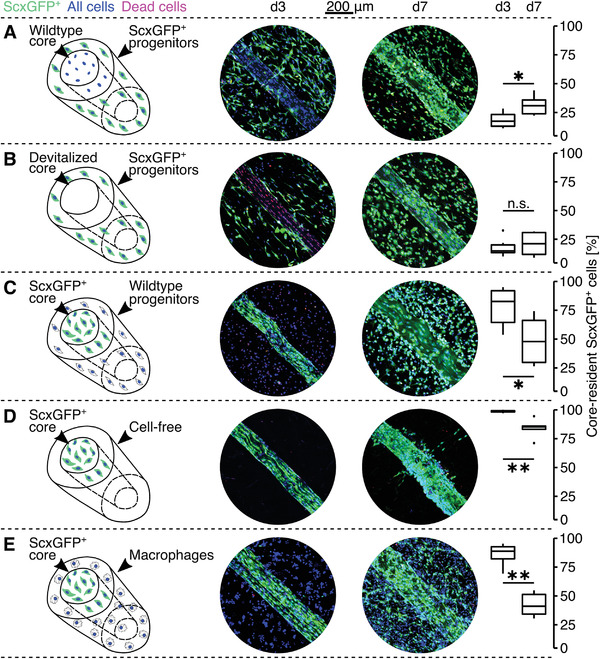
Scx‐lineage cell migration across the artificial tissue–matrix interface in tenostructs. Representative fluorescence microscopy images and migration analysis of A) a wild‐type tendon core explant embedded with extrinsic progenitors from ScxGFP^+^ mice (green), B) a devitalized, wild‐type tendon core explant embedded with extrinsic progenitors from ScxGFP^+^ mice (green), C) a tendon core explant from a ScxGFP^+^ mouse (green) embedded with wild‐type progenitors, D) a tendon core explant from a ScxGFP^+^ mouse (green) embedded in a cell‐free hydrogel, and E) a tendon core explant from a ScxGFP^+^ mouse (green) embedded with macrophages, all after 3 (d3) and 7 (d7) days of culture. All nuclei are stained with NucBlue (blue) and dead cells with EthD (red). The percentages of core‐resident/‐attracted ScxGFP^+^ cells are displayed as boxplot for each condition. *N* = 6. The upper and lower hinges correspond to the first and third quartile (25th and 75th percentile) and the middle one to the median. Whiskers extend from the upper/lower hinge to the largest/smallest value no further than 1.5 times the interquartile range. Data beyond the whiskers are depicted as dots. Results of the statistical analysis are indicated as follows: ^n.s.^ *p* > 0.05, *p < 0.05, **p < 0.01. The applied statistical test was the Wilcoxon Rank Sum.

While intact tissue–tissue interfaces and compartmentalization are a hallmark of healthy tendons, progenitors are known to home toward damaged tendon core tissue.^[^ ^12^, ^14^, ^41^, ^42^ ^]^ Since the differences in mechanical functionality of core//progenitor, core//cell‐free, and core//macrophage tenostructs disappeared when the subcompartments were cocultured physically separated, we next assessed the role of cross‐compartment migration patterns in our tenostructs, starting with Scx‐lineage cells (**Figure** **4**).

In tenostructs with extrinsic progenitors isolated from ScxGFP^+^ mice (green) and a wild‐type tendon core explant, cross‐compartmental migration led to a significant increase of core‐resident ScxGFP^+^ cells (in percentage of total ScxGFP^+^ cells) from a median (IQR) of 17.45 (10.16)% at d3 to 30.89 (12.00)% at d7 (Figure 4A). In comparison, we found reduced, nonsignificant cross‐compartmental, core‐directional migration in tenostructs containing a devitalized tendon core explant with core‐resident ScxGFP^+^ cells increasing from 15.16 (6.7)% to 21.43 (18.91)% (Figure 4B). Meanwhile, in tenostructs with extrinsic wild‐type progenitors and a ScxGFP^+^ tendon core explant, ScxGFP^+^ core‐resident tenocytes also populated the artificial extrinsic compartment, leading to a significant relative decrease of core‐resident ScxGFP^+^ tenocytes from a median (IQR) of 82.64 (27.5)% at d3 to 47.88 (36.44)% at d7 (Figure 4C). Similarly, the relative number of ScxGFP^+^ core‐resident tenocytes also decreased from 98.81 (1.04)% to 85.14 (2.82)% in core//cell‐free tenostructs and from 88.8 (10.49)% to 41.05 (17.1)% in core//macrophage tenostructs. In summary, a stressed tendon niche seems to stimulate bidirectional, cross‐compartmental cell migration in both core‐resident, Scx‐lineage tenocytes surrounded by an artificial (cell‐free/progenitor/macrophage) extrinsic compartment as well as extrinsic hydrogel‐embedded progenitors.

### 2.5 The Cellular Compositions of Tenostruct Subcompartments Shift Over Time

**Figure 5:**
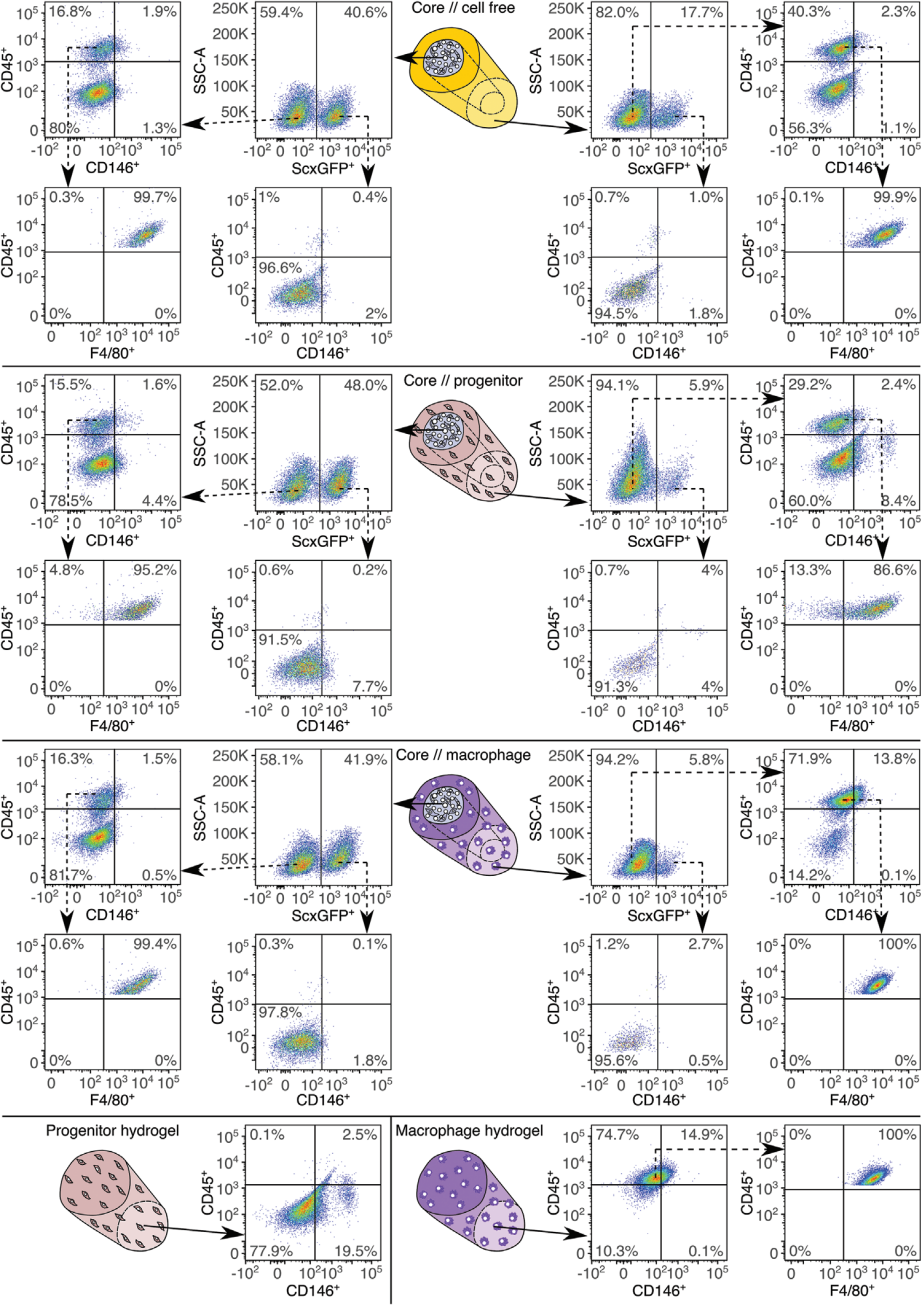
Expression of lineage markers in tenostruct subcompartments after 7 days in culture. Flow cytometric analysis of digested tendon core explants from ScxGFP^+^ mice, their cocultured extrinsic compartments, and progenitors/macrophages cultured in single‐culture control hydrogels. Assessed markers include ScxGFP, the progenitor marker CD146, the hematopoietic lineage marker CD45, and the macrophage marker F4/80.

The migration patterns of Scx‐lineage cells indicated a dynamic compartmental tissue–tissue interface, as further evidenced by light‐microscopy images of tenostructs over time (Figure S3B, Supporting Information). To deepen our understanding of this dynamic and its effects on tenostruct behavior, we assessed the cellular composition of the tenostruct subcompartments as well as the hydrogel culture controls after 7 days using flow cytometry (**Figure** **5**). Trying to distinguish between core‐derived and extrinsic cell populations, we used core explants from ScxGFP^+^ mice and extrinsic cell populations from wild‐type mice.

We found that only 40% of core‐resident cells in cultured core//cell‐free tenostructs were Scx‐lineage cells, which were also negative for both CD45 and CD146. Around 17% of the remaining core‐resident cells were of hematopoietic origin (CD45^+^) and expressed the macrophage marker F4/80. The percentage of Scx‐lineage cells among the outward migrated core cells was even lower (17.7%), and they were also negative for both CD45 and CD146. Among migrated cells, those of hematopoietic origin expressing F4/80 were more prominent (40.3%).

The core population composition in core//progenitor tenostructs was comparable to that of core//cell‐free tenostructs, albeit with a slightly higher percentage of CD146^+^ cells in both non‐Scx‐ and Scx‐lineage cells (6% and 7.9%, respectively). The percentage of ScxGFP^+^ cells in the extrinsic compartment of the core//progenitor tenostruct (5.9%) was lower than in the initially cell‐free extrinsic compartment. Combined with insights gained from the microscopy images of ScxGFP^+^ core explants (Figure 4C–E), this percentage difference should be explained by increased dilution with the seeded extrinsic wild‐type populations, and not reduced outward migration of Scx‐lineage cells in core//progenitor tenostructs. Interestingly, the percentage of CD146^+^ progenitors decreased in both the extrinsic compartment of the core//progenitor tenostructs (10.8%) as well as the control progenitor hydrogels (19.5%) compared to the seeded cells (Figure S1B, 78.7%, Supporting Information), and approached values reported for freshly digested Achilles tendons. Possible underlying mechanisms include CD146^+^ progenitor differentiation and higher proliferation rates of other present cells (hematopoietic‐ and Scx‐lineage), as we again found a hematopoietic, F4/80^+^ cell population. This population was only present in the extrinsic compartment of core//progenitor tenostructs (29.2%) and not in the control hydrogel (0.1%).

The core populations from core//macrophage tenostructs were comparable to those of the core//cell‐free tenostructs and differed from the core//progenitor tenostructs only by lacking a CD45^−^CD146^+^ population. The extrinsic compartment of core//macrophage tenostructs mainly contained hematopoietic cells expressing F4/80, likely comprising both the bone‐marrow derived macrophage population initially seeded in the extrinsic compartment as well as the hematopoietic population that migrated from the core. The percentage of CD45^+^F4/80^+^ cells in the macrophage control hydrogel was even higher, indicating that there was at least one non‐Scx‐lineage, non‐hematopoietic cell population in core//macrophage tenostructs that migrated outward from the core.

### 2.6 The Extrinsic Compartment of Core//Progenitor Tenostructs Features Increased Expression of Proinflammatory and Catabolic Markers

**Figure 6:**
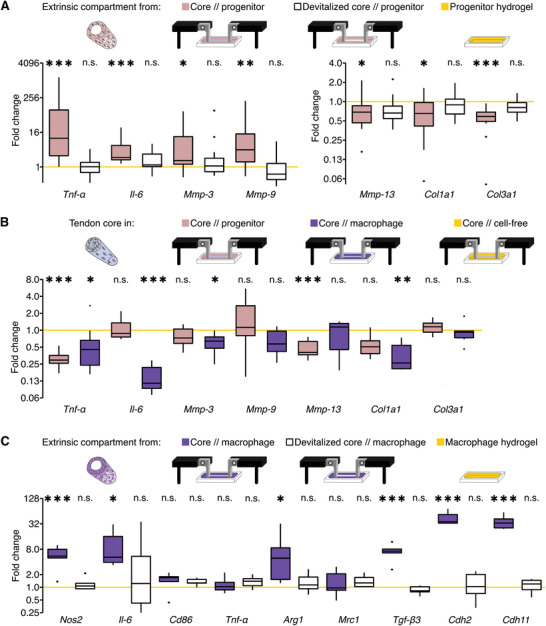
Gene‐level changes in the subcompartments of engineered tenostructs at day 7. A) Gene expression of *Tnf‐α*, *Il‐6*, *Mmp‐3*, *Mmp‐9*, *Mmp‐13*, *Col1a1*, and *Col3a1* in the extrinsic compartment of a core//progenitor tenostruct (beige) or of a core//progenitor tenostruct with a devitalized core (white). Data are normalized and compared to expression of progenitors cultured in a collagen‐1 hydrogel (progenitor hydrogel, yellow). *N* = 14. B) Gene expression of *Tnf‐α*, *Il‐6*, *Mmp‐3*, *Mmp‐9*, *Mmp‐13*, *Col1a1*, *and Col3a1* in tendon core explants surrounded by a hydrogel initially seeded with progenitors (core//progenitor, beige) or bone‐marrow derived macrophages (core//macrophage, violet). Data are normalized and compared to tendon core explants surrounded by an initially cell‐free hydrogel (core//cell‐free, yellow line). *N* = 6. C) Gene expression of *Nos2*, *Il‐6*, *Cd86*, *Tnf‐α*, *Arg1*, *Mrc1*, *Tgf‐β3*, *Cdh2*, and *Cdh11* in the extrinsic compartment of a core//macrophage tenostruct (violet) or of a core//macrophage tenostruct containing a devitalized tendon core explant (white). Data are normalized and compared to the gene expression of macrophages cultured in a control hydrogel (macrophage hydrogel, yellow). *N* = 6. Boxplots: the upper and lower hinges correspond to the first and third quartile (25th and 75th percentile) and the middle one to the median. Whiskers extend from the upper/lower hinge to the largest/smallest value no further than 1.5 the interquartile range. Data beyond the whiskers are depicted as dots. Results of the statistical analysis are indicated as follows: ^n.s.^ *p* > 0.05, *p < 0.05, **p < 0.01, ***p < 0.001. The applied statistical tests were ANOVA followed by Tukey Post‐Hoc.

The observed cell migration and the breakdown of core//cell‐free and core//progenitor tenostruct mechanical integrity suggested changes in the matrix‐remodeling complex of compartmental cell populations. Therefore, we analyzed gene‐level changes of inflammatory mediators and the tendon matrix remodeling complex (**Figure** **6**A,B).

Indeed, *Tnf‐α*, *Il‐6*, *Mmp‐3*, and *Mmp‐9* were all significantly increased in extrinsic progenitor compartments cocultured with a stressed tendon core explant (beige) compared to those cocultured with a devitalized core explant (white), and to progenitor hydrogel control culture (yellow). Both collagen‐1 (*Col1a1*) and collagen‐3 (*Col3a1*) expression were significantly reduced. By contrast, extrinsic progenitor compartments cocultured with a physically separated core explant did not increase expression of *Tnf‐α* and displayed a reduced but still significant upregulation of *Il‐6*, *Mmp‐3*, and *Mmp‐9* (Figure S5A, core/–/progenitor, Supporting Information).

Interestingly, core explants did not change their ECM and inflammatory gene‐expression (Figure 6B) when cocultured with an extrinsic progenitor compartment (core//progenitor, beige) compared to core explants cultured in a cell‐free hydrogel (core//cell‐free, yellow), except for a downregulation of *Tnf‐α* and *Mmp‐13*. This downregulation of *Tnf‐α* was not observed in core explants cocultured with a physically separated extrinsic progenitor compartment (Figure S5B, core/–/progenitor, Supporting Information).

### 2.7 Gene‐Level Changes in the Extrinsic Compartment of Core//Macrophage Tenostructs Indicate Macrophage Specialization and Macrophage–Fibroblast Interactions

Looking at the gene expression of core explants in nondegrading core//macrophage tenostructs (Figure 6B, violet), we found a significant decrease in the inflammatory mediators *Tnf‐α* and *Il‐6*, the matrix‐metalloprotease *Mmp‐3*, and the ECM protein *Col1a1* compared to core explants in core//cell‐free tenostructs (yellow). In core explants cocultured with physically separated extrinsic macrophage compartments, these differences disappeared, and mechanical degradation resumed along with elevated *Tnf‐α*, *Il‐6*, and *MMP‐3* gene expression (Figure S5B, core/–/macrophage, Supporting Information).

Meanwhile, extrinsic macrophage compartments cocultured with a stressed tendon core explant (Figure 6C, violet) showed upregulation of both the M1‐like marker *Nos2* as well as the M2‐like marker *Arg1* compared those cocultured with a devitalized core explant (white) and to macrophage hydrogel control culture (yellow). Furthermore, we found significant upregulation of *Tgf‐β3*, an M2‐like marker and signaling factor known to activate fibroblasts into repair‐competent myofibroblasts, and the macrophage–fibroblast interaction markers *Cdh2* and *Cdh11*, both downstream effectors of and signaling‐enhancers for *Tgf‐β3*. Extrinsic macrophage compartments cultured physically separated from the core explant did not upregulate *Tgf‐β3*, and the specialization (*Nos2*, *Arg1*) and fibroblast interaction (*Cdh2, Cdh11*) markers only to a lesser extent (Figure S5C, core/–/macrophage, Supporting Information).

### 2.8 Secreted IL‐6, TNF‐*α*, and MMP‐9 Predict Tenostruct Degradation

**Figure 7:**
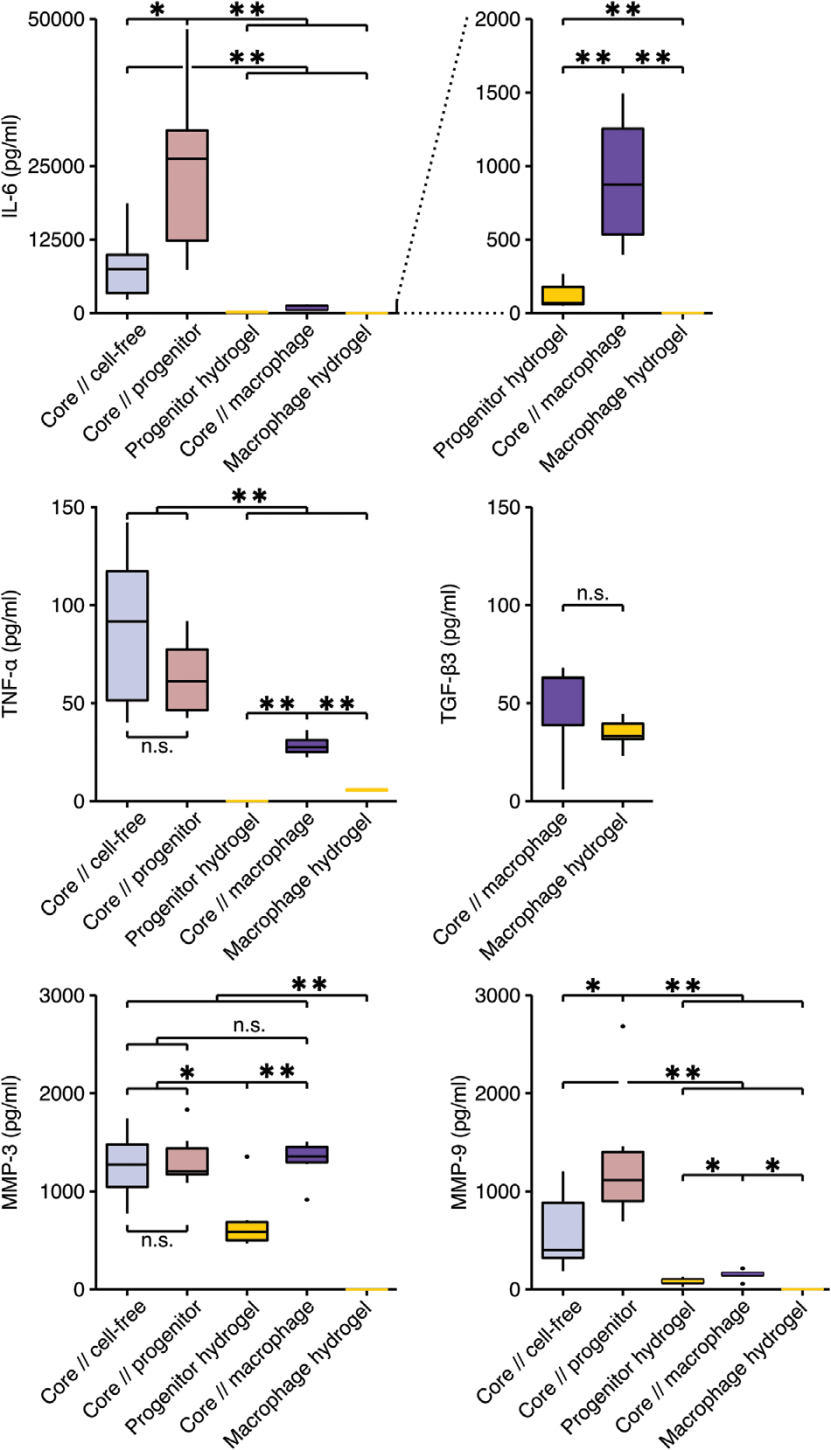
Detection of protein level differences in supernatants obtained from different tenostructs or control hydrogels at day 7. Concentration (pg mL^−1^) of IL‐6, TNF‐*α*, TGF‐*β*3, MMP‐3, and MMP‐9. *N* = 6. Boxplots: the upper and lower bounding boxes correspond to the first and third quartile (25th and 75th percentile) and the middle bar to the median. Whiskers extend from the upper/lower hinge to the largest/smallest value no further than 1.5 the interquartile range. Data beyond the whiskers are depicted as dots. Results of the statistical analysis are indicated as follows: ^n.s.^ *p* > 0.05, *p < 0.05, **p < 0.01. The applied statistical test was the Wilcoxon Rank Sum.

To assess effects of subcompartmental gene expression on tenostruct behavior, we measured the concentration of inflammatory mediators and catabolic enzymes in the supernatant of tenostructs and their hydrogel controls (**Figure** **7**). We found IL‐6 and MMP‐9 concentrations to be significantly increased in the fast‐degrading core//progenitor tenostructs compared to the slower‐degrading core//cell‐free tenostructs. Also, concentration levels of IL‐6, TNF‐*α*, and MMP‐9 were significantly higher in the degrading core//progenitor and core//cell‐free tenostructs compared to the nondegrading core//macrophage tenostructs. Surprisingly, we found no differences in MMP‐3 concentration between the tenostructs and only a trending difference in TGF‐*β*3 concentration between core//macrophage tenostructs and the macrophage control hydrogels. Overall, the concentrations of almost all assessed proteins were substantially lower in the control hydrogels compared to the tenostructs.

## 3 Discussion

Tendon disorders are often a complex pathology with a multifactorial etiology.^[^ ^4^, ^43^ ^]^ Due to our lacking understanding of this complexity, current treatment options often fail to restore tissue structure and function. A lack of adequate model systems to study tendon tissue biology and pathology is partly to blame for this. While in vitro 2D cell culture models are too simplistic, in vivo models are cumbersome and slow in separating cause–effect relationships and specific multicellular interactions.^[^ ^34^, ^35^ ^]^ In vitro 3D model systems alleviate some of these problems, as they provide a tractable cell microenvironment that retains and mimics key features of the cellular in vivo microenvironment.^[^ ^8^, ^36^ ^]^ Unfortunately, homeostasis of full tendon organ explants is difficult or even impossible to viably maintain, with tissue extraction compromising the extrinsic compartment/peritendon which is supposed to be heavily involved in repair processes.^[^ ^14^, ^18^, ^21^, ^22^, ^23^, ^24^ ^]^ To address this, we present a novel hybrid hydrogel‐explant model system (which we coin a “tenostruct”) that comprises a mechanically tractable tendon core fascicle explant with preserved native pericellular matrix and a hydrogel seeded with progenitors or bone‐marrow derived macrophages to model the extrinsic tendon compartment where such cells are thought to reside or be delivered to.

Like in vivo tendon, tenostruct mechanical properties are determined by the tendon core explant. Despite an initial volumetric mismatch between the simulated intrinsic and extrinsic compartments that is deliberately accepted to ensure complete explant embedding, radial contraction and compaction of the extrinsic compartment rapidly reproportion the tenostruct. During this process, extrinsic tendon progenitor cells were observed to align with the direction of static, uniaxial mechanical loading imposed by mechanical clamping. This alignment was not observed in the also longitudinally contracting, freefloating control hydrogels, suggesting that anchored attachments points are vital to achieve a biofidelic 3D tenostruct.

When the tenostruct system was cultured within a biochemically/mechanobiologically stressed niche (with elevated tissue oxygenation, temperature, glucose‐levels, growth factor concentration through serum supplementation, and altered loading through explantation),^[^ ^27^, ^44^ ^]^ we found prodegenerative core‐progenitor cross‐compartmental interactions evidenced by decreased mechanical properties of the tendon core and increased immunomodulatory/catabolic gene expression in the extrinsic progenitor compartment. Migration patterns of extrinsic Scx‐lineage cells indicated their activation and recruitment to the stressed and underloaded tendon core, an in vitro behavior consistent with a major hallmark of pathologies affecting tendon and other musculoskeletal tissues.^[^ ^14^, ^21^, ^45^, ^46^, ^47^, ^48^ ^]^

While the extrinsic progenitor compartment accelerated core degeneration, the underlying tissue breakdown was already detectable in core//cell‐free tenostructs and is therefore probably core‐induced. The major differences in core gene expression signatures between degrading core//progenitor, core//cell‐free tenostructs, and nondegrading core//macrophage tenostructs were increases in core *Il‐6*, *MMP‐3*, and *Col1a1*. Flow cytometric analysis of core explants and the extrinsic compartments of core//cell‐free and core//progenitor tenostructs revealed a surprisingly high percentage of cells expressing the hematopoietic marker CD45 alongside the established macrophage marker F4/80. Also, core‐resident “tenophage” or “tenoclast” populations have recently been gaining more attention as potential role players in early tendinopathy, especially in combination with increased expression of *Tnf‐α, Il‐6*, *Mmp‐3*, and *Mmp‐9*.^[^ ^44^, ^46^, ^49^, ^50^, ^51^, ^52^ ^]^ If we complement our flow cytometry data with the gene‐level increase of these markers in the extrinsic compartment of rapidly degrading core//progenitor tenostructs, (initially core‐resident but outward‐migrating) tenophages are likely to be involved in the observed core explant degeneration.^[^ ^46^ ^]^

Furthermore, secretome levels of IL‐6, TNF‐*α*, and MMP‐9 predicted the degradation speed of tenostructs and were lowest in nondegrading core//macrophage tenostructs. Meanwhile, the stressed core induced both M1‐ and M2‐like differentiation in the extrinsic compartment of core//macrophage tenostructs, with different subpopulations perhaps emerging for purposes of an effective repair response.^[^ ^49^, ^53^ ^]^ This response could be mediated through *Tgf‐β3*, which is typical for M2‐like, proresolving macrophages.^[^ ^54^ ^]^ In combination with the macrophage–fibroblast cell adhesion molecules/cadherins (Cdh11 and Cdh2) also upregulated in the extrinsic compartment of core//macrophage tenostructs, macrophage‐produced *Tgf‐β3* can transform tendon fibroblasts into extracellular matrix‐secreting myofibroblasts, which represents a critical step in wound healing and tissue repair.^[^ ^45^, ^55^, ^56^, ^57^ ^]^

In tenostructs containing a devitalized core explant or in physically separated coculture, the differences in mechanical degradation rate disappeared, and the changes in gene expression became less pronounced. This further highlights the importance of direct cross‐compartmental signaling but could also inform future experiments about the role of paracrine signaling or that of necrotic factors stemming from a devitalized core.

In summary, our tenostruct model system captures multiple key processes of tendon wound healing and repair: disturbed tissue–tissue interfaces, outward deployment of core‐resident tenophages, recruitment of extrinsic CD146^+^ progenitor and proliferating Scx‐lineage cells to the stressed tendon core,^[^ ^12^, ^14^, ^41^, ^42^ ^]^ and macrophage–fibroblast interactions.^[^ ^58^, ^59^ ^]^ It is also modular, allowing postculture isolation of the tendon core explant from the extrinsic compartment and therefore compartment‐specific analysis of crosstalk and cellular behavior. Since the in vivo paratenon contains a more diverse set of cellular subpopulations, future studies could gradually integrate these (even genetically modified) populations while using advanced downstream readout methods like single‐cell RNA‐sequencing to identify the populations dominating the tenostruct response to a more diverse set of stressors including structural microdamage. This opens the possibility for focused study on mechanisms of tendon disease pathogenesis, including a better understanding of the axis of structural core damage, recruitment of extrinsic cell populations, and temporary core vascularization. Here, another key advantage of the tenostruct model is that it allows application of mechanical, damage‐inducing forces in a precisely defined biochemical niche. Since the interfascicular matrix of tendon consists mainly of collagen‐1,^[^ ^60^, ^61^ ^]^ we opted for an extrinsic collagen‐1 hydrogel model in these first, proof‐of‐principle studies. While the interfascicular matrix is substantially more complex than modeled in our studies,^[^ ^17^ ^]^ the modular nature of our tenostructs allows increasing matrix complexity to be investigated, such as elaboration of the basic collagen‐1 hydrogel to include collagen‐3 and/or fibronectin.

## 4 Conclusion

Tendon related pathologies represent roughly a third of all musculoskeletal clinical visits. We present a tissue engineered model system that captures key features and characteristics of tendon pathology, featuring alterations in the tissue‐remodeling complex and immunomodulatory activity, consequential tendon core degeneration, increased progenitor recruitment to the degenerating tendon core, and immune system mitigation of tendon core tissue turnover. These findings combined with the flexibility gained from a modular construction suggest that this tenostruct platform may provide a useful and powerful tool for parametric study of tissue crosstalk in tendon disease, injury, and repair.

## 5 Experimental Section

### Mouse Tissue Harvest

Mouse tissue components were harvested from 12 to 16‐week‐old female B6/J‐Rj (wild‐type) and Tg(Scx‐GFP)1Stzr (ScxGFP) mice within 2 h after the sacrifice of the animal. Harvested tissue components included the mouse tail tendon fascicle explants, the mouse Achilles tendon derived CD146^+^ progenitors, and the bone‐marrow derived monocytes. All experiments were approved by the responsible authorities (Canton Zurich, license number ZH239‐17 and ZH104‐18).

### Fascicle Isolation, Preparation, and Storage

The mouse tail tendon fascicle explants (core explants) were gently extracted and cut into 30 mm long pieces, which were kept in culture medium (DMEM/F12 GlutaMAX with 1% penicillin/streptomycin (v/v), 200 µM l‐ascorbic acid, 10% fetal bovine serum (v/v)) until the start of the experiment. Meanwhile, the devitalized control core explants stored frozen at −20 °C inside the tails were thawed, extracted, and cut. For all core explants, their diameter was measured at three different places along each explant, the mean diameter was calculated, and explants with a mean diameter below 100 µm or above 150 µm were excluded. All medium components were purchased from Sigma‐Aldrich, except for the ascorbic acid (Wako Chemicals) and the collagenase (ThermoFisher).

### CD146^+^ Progenitor Isolation and Culture

First, the skin spanning mouse feet and hindlimbs was sterilized and then removed with 80% ethanol in water. The mouse Achilles tendons were separated from the calcaneus and the calf muscles using a scalpel and placed in PBS for washing and removal of leftover nontendon tissue. For the digestion, 2–6 Achilles tendons were pooled, 10 mL digestion medium (DMEM/F12 GlutaMAX with 1% penicillin/streptomycin (v/v), 1% Amphotericin (v/v), 2 mg mL^−1^ collagenase I (17100‐017, Gibco)) was added and they were incubated overnight at 37 °C under constant agitation/turning. Then, the digested solution was centrifuged for 5 min at 500 g and the pellet was resuspended in culture medium. This step was repeated once before transferring the cell suspension to a T25 cell culture flask, which was then put into an incubator (37 °C, 20% O_2_). The cell culture medium was changed once a week and the cells split once they reached 80% confluency. For the actual experiments, progenitors were only used between P2 and P4, for which CD146^+^ expression using fluorescence microscopy and flow cytometry was quantified. All medium components were purchased from Sigma‐Aldrich, except for the ascorbic acid (Wako Chemicals).

### Fluorescence Microscopy for CD146‐Expression

Fluorescence microscopy was first used to compare CD146‐expression in mouse Achilles tenocytes at different passage numbers and in freshly isolated mouse tail tendon fascicles. To do so, isolated mouse Achilles tenocytes were cultured in stressed niche conditions (37 °C, 20% O_2_) with standard culture medium in two separate wells of a 2D tissue culture plate. After reaching 80% confluency, the cells of one well were split 1:5. Once the cells in the second well reached 100% confluency, they were washed with PBS and fixated with 4% paraformaldehyde (Roti‐Histofix, Karlsruhe) for 10 min at room temperature. Then, the cells were washed again with PBS (three times) and kept in PBS at 4 °C until staining. The splitting and fixation procedures were repeated with the new wells in the same manner until the cells reached P4 and the same fixation protocol was used to prepare mouse tail tendon fascicles/core explants. For the immunostaining, the cells/core explants were incubated for 60 min with 3% bovine‐serum albumin (BSA, Merck Millipore) in PBST (PBS + 0.1% Tween 20) at room temperature to block unspecific antibody‐binding. Next, the blocking solution was replaced with the primary staining solution consisting of the primary antibody against CD146 (polyclonal, bs‐1618R‐TR from Bioss ANTIBODIES) dissolved 1:200 in PBST with 1% BSA. The fixated cells/core explants were then incubated overnight in a humidified chamber at 4 °C together with the primary staining solution. After washing the now stained cells/core explants with PBS (three times, 5 min each) and adding the secondary staining solution, consisting of the secondary antibody (Alexa Fluor 488, Donkey Anti‐Rabbit IgG from ThermoFisher) diluted 1:1000 in PBST with 1% BSA, they were incubated in a dark, humidified chamber for 1 h at room temperature. After another washing step with PBS (three times, 5 min each, in the dark) the samples were counterstained with NucRed Live 647 ReadyProbes (R37106, Invitrogen) in PBS (1 drop in 0.5 mL). For the fluorescence microscopy, the Nikon Eclipse T*i*2 confocal laser scanning microscope with NIS‐Elements was used.

### Macrophage Isolation and Culture

Bone‐marrow derived monocytes were isolated from mouse femur and tibia. To isolate the bones, the skin spanning mouse feet and hindlimbs was decontaminated with 80% ethanol in water and then removed. Next, the leg was separated from the body and the foot by cutting at the hip and the ankle joint. Then, the muscle tissue was removed and the bones were washed in cold PBS before cutting away the epiphyses to expose the bone‐marrow. Afterward, the bone‐marrow was flushed into a 50 mL falcon tube using 10 mL warm macrophage culture medium (DMEM/F12 high glucose, 10% FBS, 1% nonessential amino acids, 1% penicillin/streptomycin). The cell suspension was then filtered (100 µm nylon filter) and centrifuged (350 g for 5 min). To remove the red blood cells, the cell pellet was resuspended in RBC lysis buffer (VWR) and the suspension centrifuged again (10 min at 350 g). Finally, the pellet was resuspended in macrophage culture medium (DMEM/F12 high glucose, 10% FBS, 1% nonessential amino acids, 1% P/S) and the cell‐suspension transferred to untreated petri dishes (5–8 Mio cell density per 100 mm culture dish). After 4 h and until d6, 20 ng mL^−1^ macrophage‐colony stimulating factor (recombinant M‐CSF, PeproTech, 315‐02) was added to initiate differentiation of the monocytes toward naïve macrophages (M0). Expression of the macrophage surface markers CD45 and F4/80 was checked using flow cytometry.

To chemically specialize naïve macrophages toward an M1‐phenotype, they were treated with 20 ng mL^−1^ interferon‐*γ* (IFN‐*γ*, Miltenyi Biotec) and 50 ng mL^−1^ lipopolysaccharides (LPS, Sigma) and the success of the chemical specialization was verified by assessing gene expression of specific surface markers (*Cd86* and *Tnf‐α*). Meanwhile, naïve macrophages were specialized toward an M2‐phenotype with 20 ng mL^−1^ of interleukin‐4 (Il‐4, Miltenyi Biotec), which was validated through analysis of surface marker gene expression (*Mrc1* and *Chi3l3*).

### Collagen Isolation

Collagen‐1 was isolated from rat tail tendon fascicles following an established protocol.^[^ ^62^ ^]^

### Fabrication of Chambers, Clamps, and Mounting Platform

To make the chambers, custom 3D‐printed molds were filled with Dragon Skin 10 Slow/1 (KauPO), which was degassed in a vacuum chamber (90 mbar) for 30 min, and let polymerize on a hot plate at 70 °C for 1 h. The clamps consisted of metal pincers (stainless steel, Supporting Information) were screwed to polyetherimide pincers holders (PEI, Supporting Information) using a mounting platform (PEI, Supporting Information).

### Hydrogel Preparation, Fascicle Embedding, and Tenostruct Culture

First, the fascicles/core explants were fixated with the custom‐made clamps at a clamp‐to‐clamp distance of 20 mm. They were placed into the molds lining the bottom of the custom‐made culture chambers, and then covered with 200 µL of the collagen hydrogel (cell‐laden or cell free) (Figure 1). The progenitor or macrophage laden control hydrogels were cast in same‐sized silicone molds (Figure S3A,D, Supporting Information). To get the physically separated coculture (core/–/cell free, core/–/progenitor, core/–/macrophage), the core explants were clamped slightly higher in the clamps, so that they were not in contact with the cast hydrogel (Figure S5, Supporting Information). Each hydrogel comprised 10 µL PBS (20×), 1.28 µL of 1 m NaOH (125×), 8.72 µL (23×) double‐distilled water (ddH_2_0), 80 µL collagen‐1 (2.5× or 1.6 mg mL^−1^), and 100 µL cell suspension (2×). For the core//progenitor tenostructs, the cell suspension consisted of CD146^+^ progenitors (from mouse Achilles tendons) redissolved in casting medium (DMEM/F12 GlutaMAX with 1% penicillin/streptomycin (v/v), 10% fetal bovine serum (v/v)) at a final concentration of 250 000 cells mL^−1^. For the core//macrophage tenostructs, the cell suspension consisted of naïve (M0) bone‐marrow derived macrophages also redissolved in casting medium at a final concentration of 370 000 cells mL^−1^. To facilitate the casting process, ensure hydrogel homogeneity and prevent premature crosslinking, the components for all hydrogels were kept on ice in one of two premixes. The first premix consisted of NaOH, ddH_2_0, and PBS, the second of collagen‐1 and the cell suspension. After 50 min polymerization at 37 °C, the hydrogel was sufficiently stable, and 2 mL of either standard tenocyte culture medium (core//progenitor tenostruct) or macrophage culture medium supplemented with 20 ng mL^−1^ M‐CSF (core//macrophage tenostructs) could be added. The tenostructs were then cultured under stressed niche conditions (37 °C, 20% O_2_) until the defined timepoint, with one media change per week.

### Viability and Morphology Analysis

At day 3 and day 7, the tenostructs were removed from the clamps and washed with PBS. Then, the tenostructs were stained with ethidium homodimer (EthD‐1, Sigma‐Aldrich, 2 × 10^−3^ m stock in DMSO) diluted in PBS to 4 × 10^−6^ m in PBS for 20 min at 37 °C, washed with PBS (three times, 10 min each) and then fixated with 4% formaldehyde (Roti‐Histofix, Karlsruhe) for 20 min at room temperature. The tenostructs were washed again with PBS (three times, 10 min each) and stored in PBS at 4 °C until further usage. Shortly before the imaging, the tenostructs were placed in 0.5 ml PBS containing one drop of NucBlue Live Ready Probes Reagent (R37605, ThermoFisher) to stain the nucleus and 4 µL mL^−1^ phalloidin‐488 (Invitrogen) to stain the actin cytoskeleton for 20 min at room temperature. For the fluorescence microscopy, the Nikon Eclipse T*i*2 confocal laser scanning microscope with NIS‐Elements was used.

ImageJ 1.52h was used to quantify aspect ratio and orientation angle of extrinsic progenitors/macrophages in the control hydrogels or the core//progenitor/core//macrophage tenostructs. These values were then analyzed in RStudio 1.1.463 and the conditional mean was calculated through locally estimated scatterplot smoothing (LOESS).

### Mechanical Testing and Analysis

To measure their mechanical properties, the samples (clamped core explants/tenostructs) were mounted to the custom‐made uniaxial stretching device equipped with a 10 N load cell (KD 24s, Lorenz Messtechnik GmbH, Altdorf, Germany) (Figure S3C, Supporting Information) and operated by our own software.^[^ ^63^ ^]^ The preload was set to 0.03 N and all samples preconditioned with five stretch cycles to a strain equal to 1% of the fascicle/construct length at preload (= *L*_0_). A stretching speed of 0.1 mm s^−1^ was used for the preconditioning and all subsequent experiments. To measure the linear elastic modulus (E‐Mod) of the samples, they were stretched to up to 2% *L*_0_ (the estimated end of the linear elastic region).^[^ ^9^ ^]^ This measurement was repeated after 3 (d3), 7 (d7), 14 (d14), and 21 days (d21) to track the temporal development. Mechanical tenostruct properties were also characterized and compared to unmodified tendon core explants by dividing one fascicle into two subunits, incorporating one into a cell‐laden collagen‐hydrogel and measuring the mechanical properties of both. After the preconditioning, the paired samples were stretched to 15% *L*_0_ while recording the stress–strain relationship. For all mechanical experiments, Matlab R2017a and RStudio 1.1.463 were used to analyze the stress–strain curve and read‐out the relevant parameters including the elastic modulus, the maximally achieved stress, and the maximally achieved strain (before sample failure).

### Cross‐Compartmental Cell Migration Analysis

Genetically modified mice expressing a green‐fluorescent variant of the tendon marker scleraxis (ScxGFP^+^) were used to follow cell migration.^[^ ^64^ ^]^ Specifically, wild‐type fascicles (alive or devitalized through repeated freezing) were embedded in hydrogels seeded with ScxGFP^+^ progenitors isolated from mouse Achilles tendons to assess ScxGFP^+^ progenitor migration toward the core. Meanwhile, ScxGFP^+^ core explants were embedded in a cell‐free hydrogel or a hydrogel seeded with progenitors/macrophages isolated from wild‐type mice to follow migration of Scx‐lineage cells from the tendon core to the extrinsic cell‐free/progenitor/macrophage compartment. For the fluorescence microscopy, the Nikon Eclipse T*i*2 confocal laser scanning microscope with NIS‐Elements was used. Using ImageJ 1.52h, ScxGFP^+^ cells were counted in the artificial extrinsic compartment or the native tendon core explant to quantify migration of Scx‐lineage cells.

### Flow Cytometry

Flow cytometry was used to quantify ScxGFP and CD146 levels in mouse Achilles tendon derived progenitors at different passages, freshly isolated mouse tail tendon fascicles, and the different tenostruct subcompartments/hydrogel controls after culture. Also, expression of the macrophage markers CD45 and F4/80 was quantified in the bone‐marrow derived macrophages after 1 week in culture and in the different tenostruct subcompartments/hydrogel controls after culture.

To get the mouse tail tendon fascicle and hydrogel‐embedded cells into suspension, multiple core explants/hydrogels were pooled and digested in 1 mL PBS with 3 mg mL^−1^ collagenase I (17100‐017, Gibco) and 4 mg mL^−1^ dispase II (D4693‐1G, Sigma) for 1 h at 37 °C under constant agitation/turning. To detach cultured mouse Achilles tendon derived progenitors and mouse bone‐marrow derived macrophages, the culture medium was removed from the T‐25 flasks. They were washed with warm PBS before adding 1 mL accutase(R) solution (Sigma‐Aldrich). After counting the cells, a maximum of 100 000 cells were distributed to each prepared vial, centrifuged, and then resuspended in one of the respective staining solutions. In the first round, CD146 expression was only assessed in freshly isolated fascicles and expanded mouse Achilles progenitors. Here, the following solutions were used: unstained control in 100 µL FACS‐buffer (PBS, 1% FBS (v/v)), isotype control in 100 µL FACS‐buffer containing 0.03 µg PE *κ* isotype ctrl antibody (Rat IgG2a, Biolegend), and 0.5 µL ZombieAqua viability staining (Biolegend), and finally the experimental group in 100 µL FACS‐buffer containing 0.03 µg PE anti‐mouse CD146 antibody (Biolegend) and 0.5 µL ZombieAqua viability staining (Biolegend). In the second round, expression of CD146, ScxGFP, CD45, and F4/80 in cultured tenostruct subcompartments/hydrogel controls, bone‐marrow derived macrophages and progenitors was assessed. Here, the cells were stained with 0.03 µg PE anti‐mouse CD146 antibody (Biolegend), 0.25 µg PerCP/Cy5.5 anti‐mouse CD45 antibody (Biolegend), 0.5 µg APC/Fire anti‐mouse F4/80 antibody (Biolegend), and 0.5 µL ZombieAqua in 100 µL FACS‐buffer.

After 30 min incubation staining at room temperature, the staining solution was diluted in 1.5 mL of FACS buffer and centrifuged for 5 min at 500 g. Then, the prepared cells were resuspended in 350 µL of FACS buffer, filtered through 35 µm nylon mesh strainer caps (Corning) to remove remaining debris, and stored at 4 °C until flow cytometric analysis. For the flow cytometric measurements, an LSR II Fortessa device was used with FACS Diva software (Becton Dickinson & Company). Finally, the data readout was analyzed using FlowJo 10.6.1 (Becton Dickinson & Company).

### Gene Expression Analysis

After 7 days of coculture, the tenostructs were removed from the clamps and the artificial extrinsic compartments separated from the tendon core explants. Pooling 2 hydrogels or 12 core explants, the samples were dissolved in GENEzol reagent (Geneaid) and the remaining extracellular matrix was broken up with metal beads in the Tissuelyser LT (QIAGEN) and through repeated pipetting with a 1 mL syringe equipped with a 21G needle. The RNA was then isolated with the PureLink RNA Micro Scale Kit (Invitrogen) according to the manufacturers protocol and RNA yield and purity were measured with the Nanodrop 1000 spectrophotometer 3.7.1 (ThermoFisher). The RNA was reverse transcribed to cDNA using the High‐Capacity RNA‐to‐cDNA Kit (Applied Biosystems) and the Eppendorf Mastercycler (Eppendorf). For qPCR, the StepOnePlus Real‐Time PCR System (Thermofisher) was used in combination with either Taqman primers (all tenocyte/progenitors samples, *Arg1*, *Nos2*, *Tgf‐β3*) normalized to the housekeeper *GAPDH* or SybrGreen primers (*Mrc1*, *Cd86*, *Tnf‐α*, *Cdh11*, *Cdh2, Chi3l3*) normalized to the housekeeper *Rpl4*.

### Secretome Analysis

Culture medium was enriched with the secretome of the different tenostructs (core//cell‐free, core//progenitor, core//macrophage) or hydrogels (progenitors, macrophages) for three days and until day 7 of the tenostruct/hydrogel culture. IL‐6, TNF‐*α*, and MMP‐9 were quantified using a custom‐made multiplex U‐PLEX for mouse biomarkers, and TGF‐*β*3 using a singleplex U‐PLEX for mouse biomarkers (both from Meso Scale Discovery) according to the manufacturer's instruction. Plates were read with the MESO Quickplex SQ120 (Meso Scale Discovery) and analyzed with Discovery Workbench 4.0.13 (https://www.mesoscale.com/en/products_and_services/software). MMP‐3 was quantified using a Mouse MMP‐3 ELISA Kit (RAB0368, Sigma‐Aldrich) according to the manufacturer's instructions. The plate was read with the Epoch Microplate Spectrophotometer (Biotek), and the data were analyzed with Microsoft Excel (https://www.microsoft.com/en‐us/microsoft‐365/excel).

### Statistical Analysis and Graphical Displays

RStudio 1.1.463 (https://rstudio.com) was used for data curation and statistical analysis. Longitudinal mechanical data was normalized to d0, core‐residing ScxGFP^+^ cells to the total number of ScxGFP^+^ cells in the image, q‐PCR data to the housekeeper and the control sample. Negative values for the elastic modulus and secretome concentrations were set to 0. Normally distributed datasets were analyzed by ANOVA followed by Tukey Post‐Hoc tests for pairwise comparisons. Otherwise, a nonparametric test (Wilcoxon Rank Sum) was applied, with the direction (less, greater, two‐sided) matching that of the data. For all tests, the level of *p*‐values was tested. The median and the interquartile range (IQR) was reported while using boxplots to represent the following data: mechanical properties of core explants and tenostructs at d0, percentage of core‐residing ScxGFP^+^ cells, gene expression fold change, and secretome concentrations. Here, the upper and lower hinges correspond to the first and third quartile (25th and 75th percentile) and the middle one to the median. Whiskers extend from the upper/lower hinge to the largest/smallest value no further than 1.5 times the interquartile range. Data beyond the whiskers are depicted as dots. To represent the longitudinal development of tenostruct mechanical properties, the mean (± sem) was displayed.

All graphical displays were made with the open‐source graphics software Inkscape 0.92.3 (www.inkscape.org) based on pdf outputs from RStudio/FlowJo 10.6.1.

## Conflict of Interest

The authors declare no conflict of interest.

## Supporting information

Supporting Information

## Data Availability

The data that support the findings of this study are available from the corresponding author upon reasonable request.
