# Supplementary material for: Extrinsic Macrophages Protect While Tendon Progenitors Degrade: Insights from a Tissue Engineered Model of Tendon Compartmental Crosstalk
Source: Adv Healthc Mater. 2021 Sep 8;10(20):2100741. doi: 10.1002/adhm.202100741 (PMC11468160; doi:10.1002/adhm.202100741)
Supplement: Supplementary file 1 — Supporting Information [file ADHM-10-2100741-s001.pdf]

© 2021 Wiley-VCH GmbH

ADVANCED HEALTHCARE MATERIALS

Supporting Information

for *Adv. Healthcare Mater.*, DOI: 10.1002/adhm.202100741

Extrinsic macrophages protect while tendon progenitors degrade – insights from a tissue engineered model of tendon compartmental crosstalk

*Tino Stauber, Maja Wolleb, Anja Duss, Patrick K. Jaeger, Irina Heggli, Amro A. Hussien, Ulrich Blache, Jess G. Snedeker**

Supporting Information

Extrinsic macrophages protect while tendon progenitors degrade – insights from a tissue engineered model of tendon compartmental crosstalk

*Tino Stauber, Maja Wolleb, Anja Duss, Patrick K. Jaeger, Irina Heggli, Amro A. Hussien, Ulrich Blache, Jess G. Snedeker**

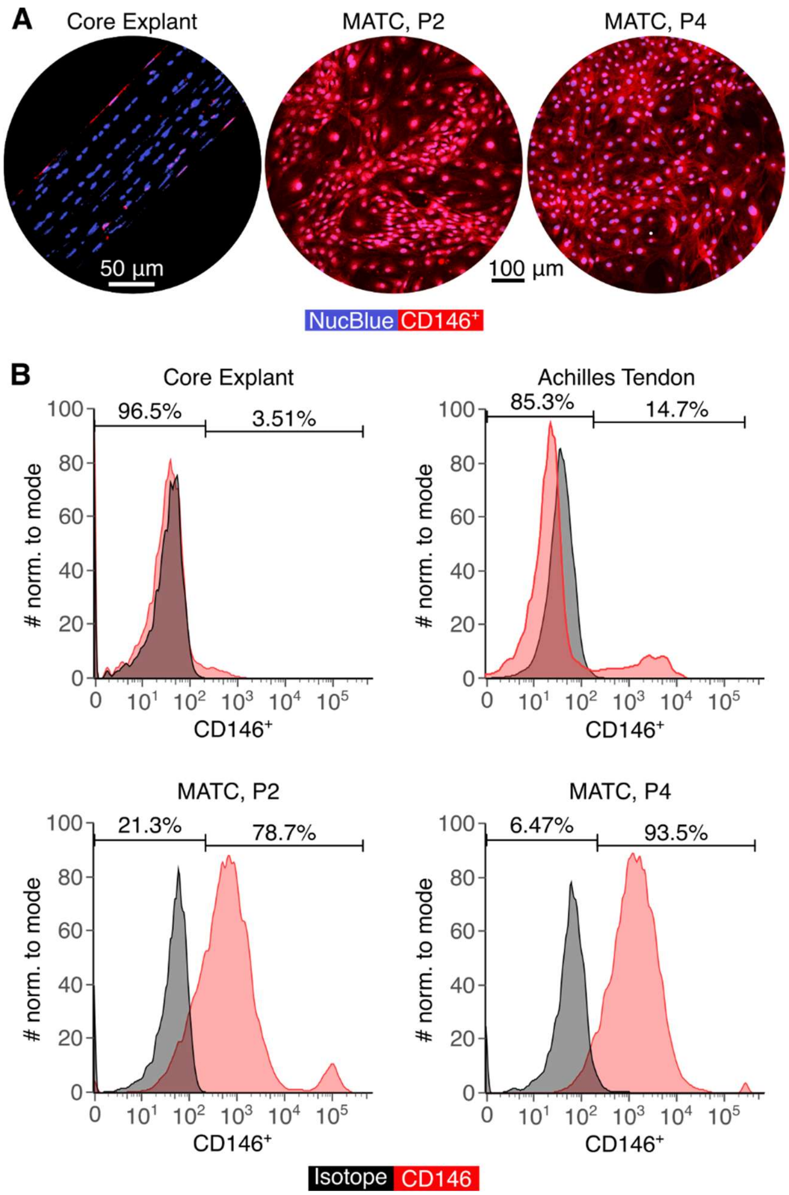

Figure S1: Expression of the CD146$^+$ progenitor marker in mouse tendon core explants, mouse Achilles tendons, and expanded tendon stromal cells derived from mouse Achilles tendons (MATC).

(A) Representative FM images of a tendon core explant and mouse Achilles tendon cells expanded to P2 & P4. Nuclei are stained with NucBlue (blue) and progenitor-like cells with a

CD146$^+$ antibody (red). (B) Flow cytometric analysis of digested tendon core explants, digested mouse Achilles tendons, and mouse Achilles tendon cells expanded to P2 & P4. Isotope and CD146$^+$ counts are normalized to modal and percentages are given for the CD146 stained group.

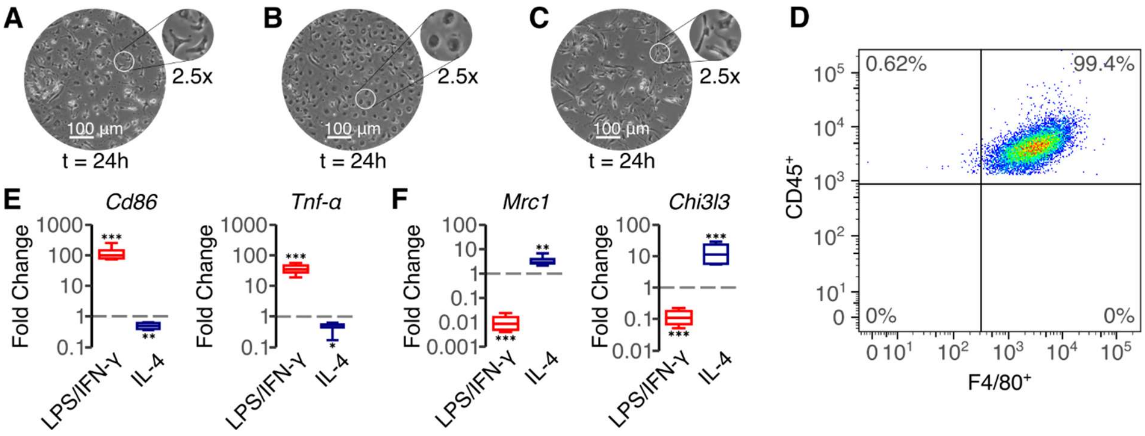

Figure S2: Chemical specialization of naïve, bone-marrow derived mouse macrophages. Representative LM images of naïve, bone-marrow derived macrophages after 24h in (A) standard macrophage culture medium, (B) standard culture medium supplemented with LPS / IFN-γ, and (C) standard culture medium supplemented with IL-4. (D) Flow cytometric analysis of cultured bone-marrow derived macrophages for CD45 and F4/80 expression. (E) M1- and (F) M2-macrophage marker expression in bone-marrow derived mouse macrophages treated with LPS / IFN-γ or IL-4, normalized to those in standard macrophage culture medium. Data are compared to macrophages cultured in standard culture medium (dashed line). N=6. Box plots: The upper and lower hinger correspond to the first and third quartile (25th and 75th percentile) and the middle one to the median. Whiskers extend from the upper / lower hinge to the largest / smallest value no further than 1.5 the interquartile range. Data beyond the whiskers are depicted as dots. Results of the statistical analysis are indicated as follows: ***p < 0.001, **p < 0.01, *p < 0.05. The applied statistical tests were ANOVA followed by Tukey Post-Hoc.

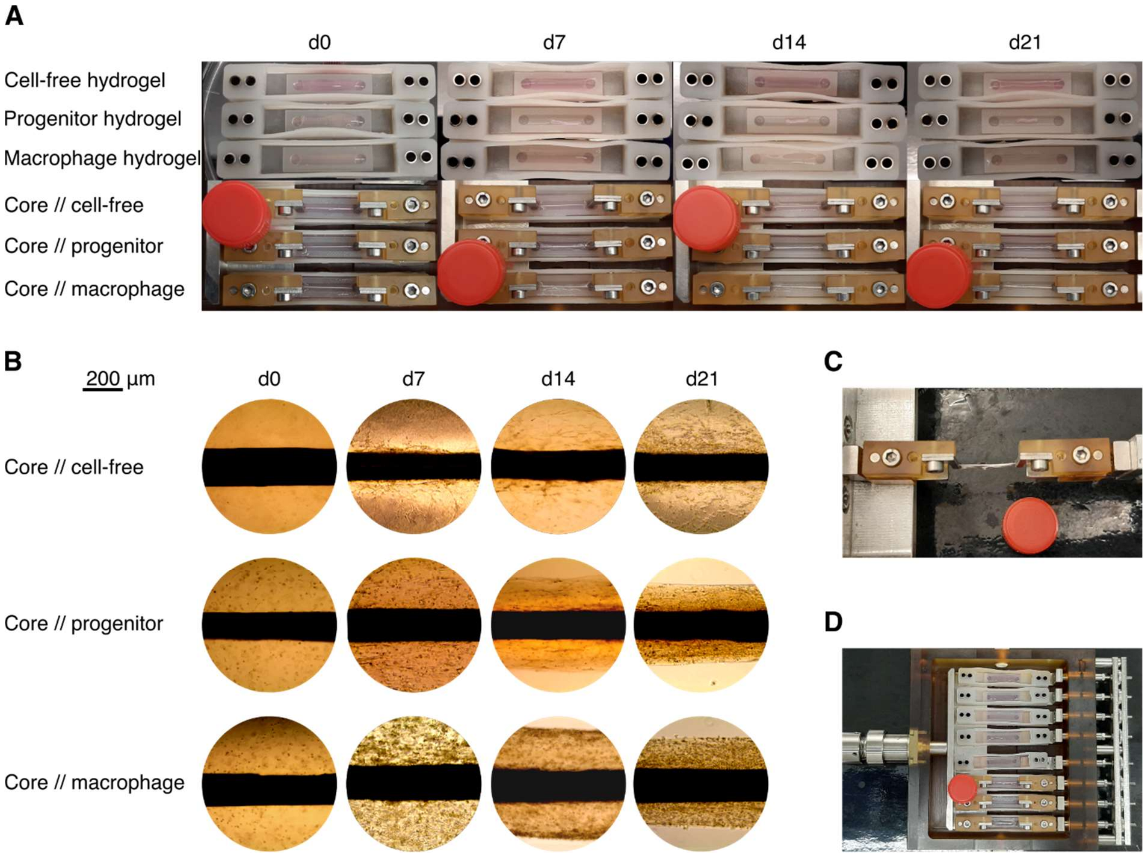

Figure S3: Tenostruct fabrication, mechanical testing, and development during culture. (A) Representative photographic images depicting the hydrogel controls and engineered tenostructs in their wells immediately after casting the hydrogel and every 7 days for 21 days. (B) LM-Images of the engineered tenostructs immediately after casting the hydrogel and every following week for 21 days. (C) Photographic image showing a clamped tenostruct mounted to the stretching device. (D) Photographic image showing the hydrogels and engineered tenostructs in the loadable bioreactor used here for static culture. Lid of a 15 ml Falcon® tube (Ø: 17 mm) used for size comparison.

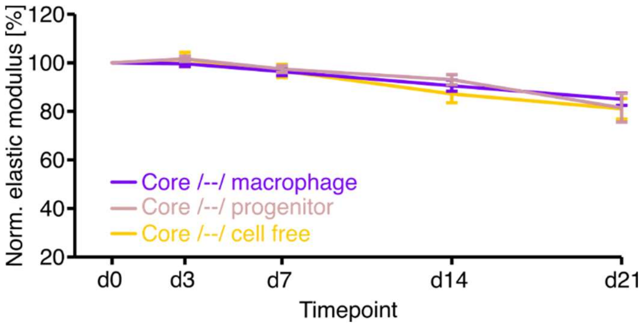

Figure S4: Mechanical properties of tendon core explants co-cultured with a spatially separated hydrogel. Linear elastic moduli of core /--/ cell free, core /--/ progenitor, and core /--/ macrophage tenostructs over a time course of 21 days. Datapoints are normalized to the initial linear elastic modulus (d0) of the sample. N=12. The data are displayed as mean (± sem).

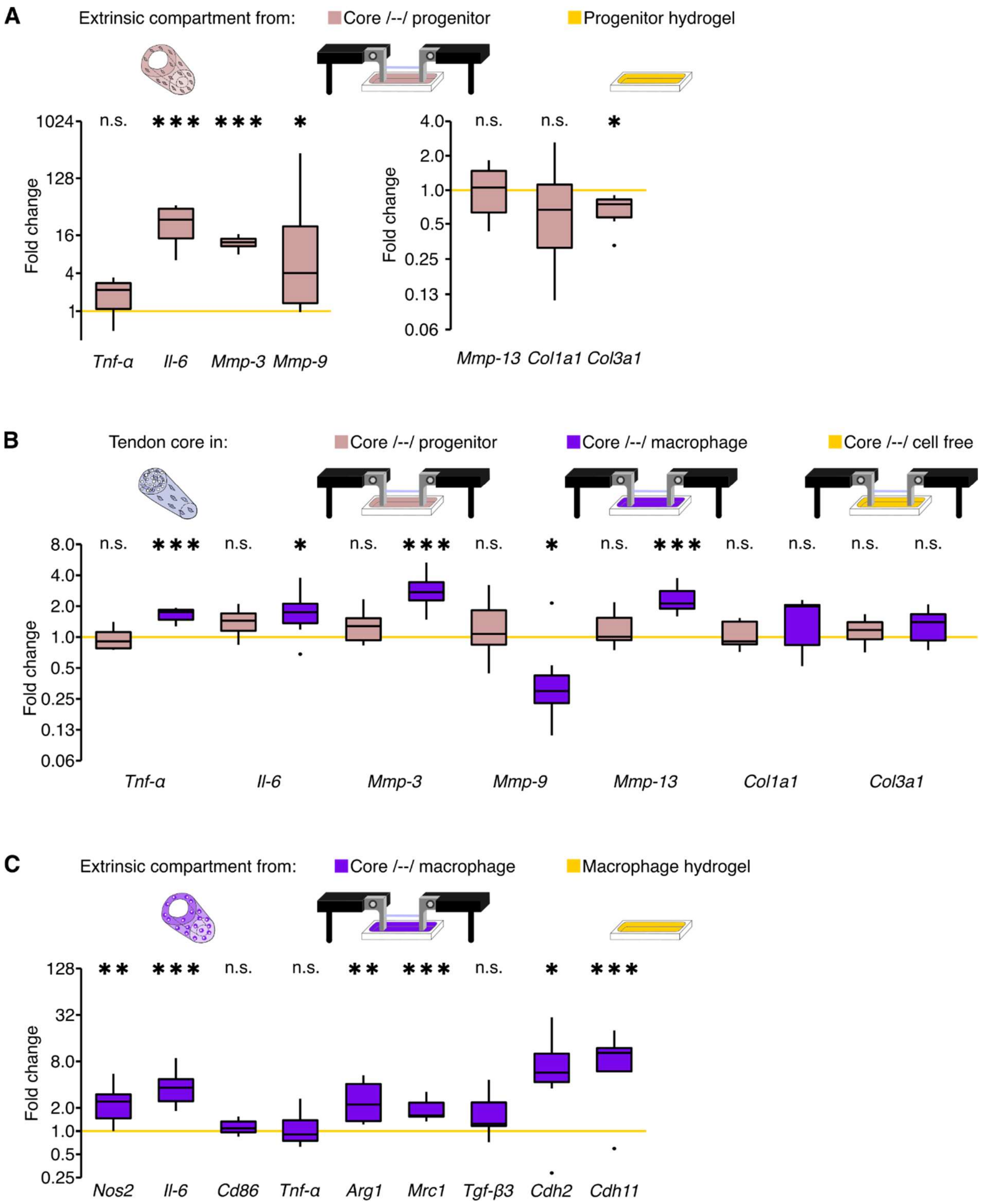

Figure S5: Gene-level changes in the spatially separated sub-compartments of engineered tenostructs at day 7. (A) Gene expression of *Tnf-α*, *Il-6*, *Mmp-3*, *Mmp-9*, *Mmp-13*, *Col1a1* and *Col3a1* by the extrinsic compartment of a core /--/ progenitor tenostruct (beige). Data are normalized and compared to expression of progenitors cultured in a collagen-I hydrogel (progenitor hydrogel, yellow). N=6. (B) Gene expression of *Tnf-α, Il-6, Mmp-3, Mmp-9, Mmp-13, Col1a1 and Col3a1* by tendon core explants co-cultured with a hydrogel seeded with progenitors (core /--/ progenitor, beige) or bone-marrow derived macrophages (core /--/ macrophage, violet). Data are normalized and compared to tendon core explants co-cultured with an initially cell free hydrogel (core /--/ cell free, yellow line). N=7. (C) Gene expression of *Nos2, Il-6, Cd86, Tnf-α, Arg1, Mrc1, Tgf-β3, Cdh2* and *Cdh11* by the extrinsic compartment of a core /--/ macrophage tenostruct (violet). Data are normalized and compared to expression

of macrophages cultured in a collagen-I hydrogel (macrophage hydrogel, yellow). N=7. Boxplots: The upper and lower hinger correspond to the first and third quartile (25th and 75th percentile) and the middle one to the median. Whiskers extend from the upper / lower hinge to the largest / smallest value no further than 1.5 the interquartile range. Data beyond the whiskers are depicted as dots. Results of the statistical analysis are indicated as follows: $^{n.s.}p > 0.05$, $^{*}p < 0.05$, $^{**}p < 0.01$, $^{***}p < 0.001$. The applied statistical tests were ANOVA followed by Tukey Post-Hoc.

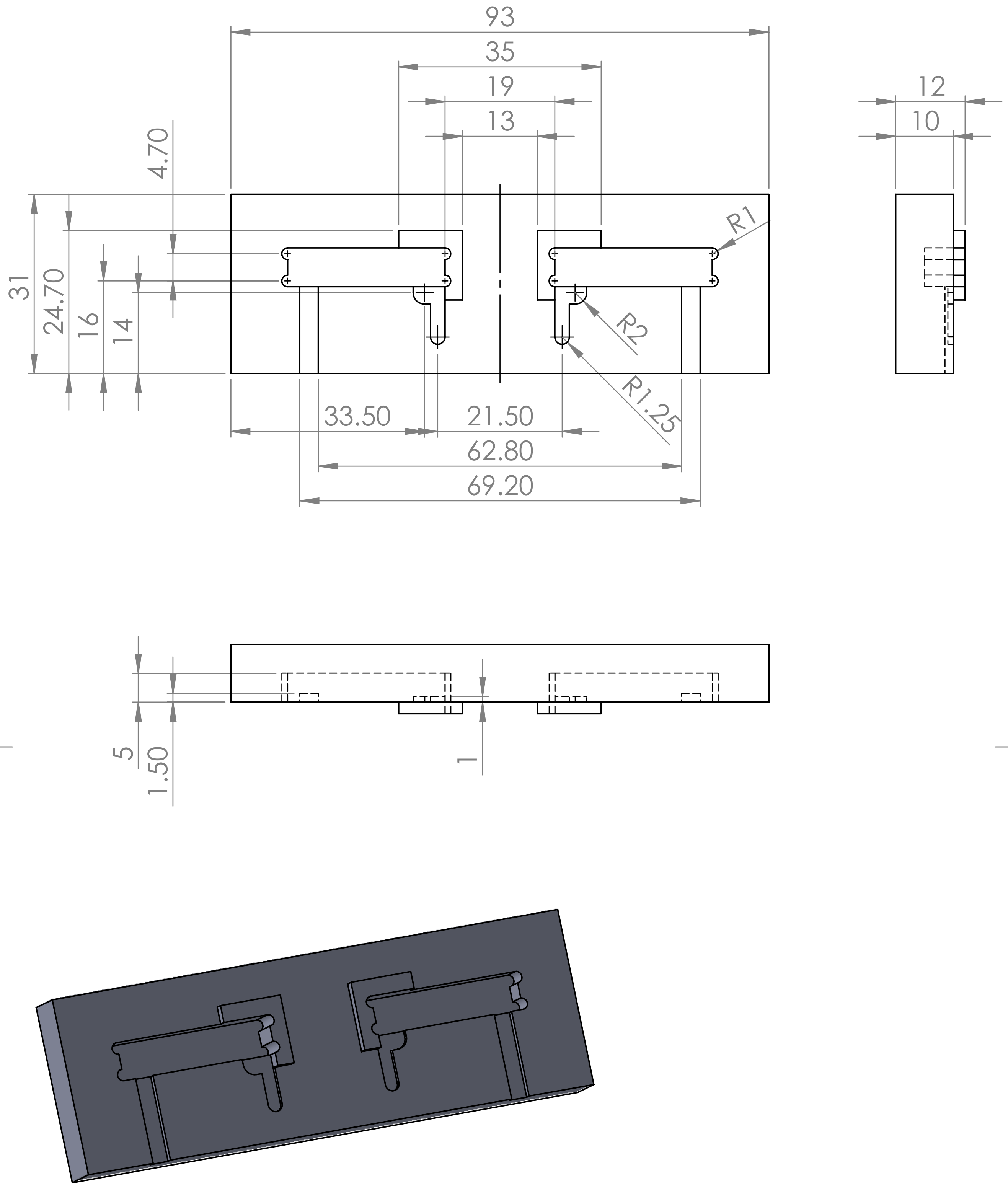

UNLESS OTHERWISE SPECIFIED:
DIMENSIONS ARE IN MILLIMETERS
SURFACE FINISH:
TOLERANCES:
LINEAR:
ANGULAR:

FINISH:

DEBURR AND BREAK SHARP EDGES

DO NOT SCALE DRAWING

REVISION

	NAME	SIGNATURE	DATE
DRAWN			
CHK'D			
APPV'D			
MFG			
Q.A			

TITLE:

MATERIAL:

DWG NO.

mounting_platform_PE

A4

WEIGHT:

SCALE:1:1

SHEET 1 OF 1

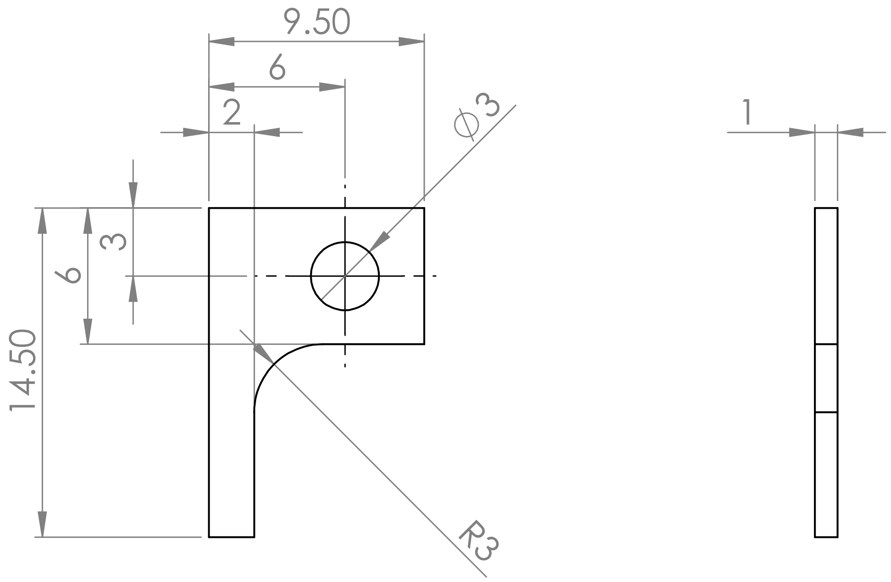

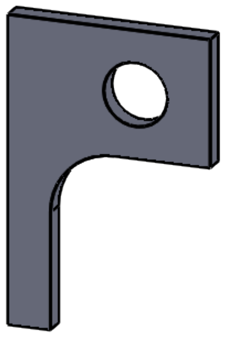

UNLESS OTHERWISE SPECIFIED:<br>DIMENSIONS ARE IN MILLIMETERS<br>SURFACE FINISH:<br>TOLERANCES:<br>LINEAR:<br>ANGULAR:	FINISH:	DEBURR AND BREAK SHARP EDGES	DO NOT SCALE DRAWING

REVISION

	NAME	SIGNATURE	DATE
DRAWN			
CHK'D			
APPV'D			
MFG			
Q.A			

TITLE:

MATERIAL:
Edelstahl

DWG NO.
pincer_short

A4

WEIGHT:

SCALE:10:1

SHEET 1 OF 1

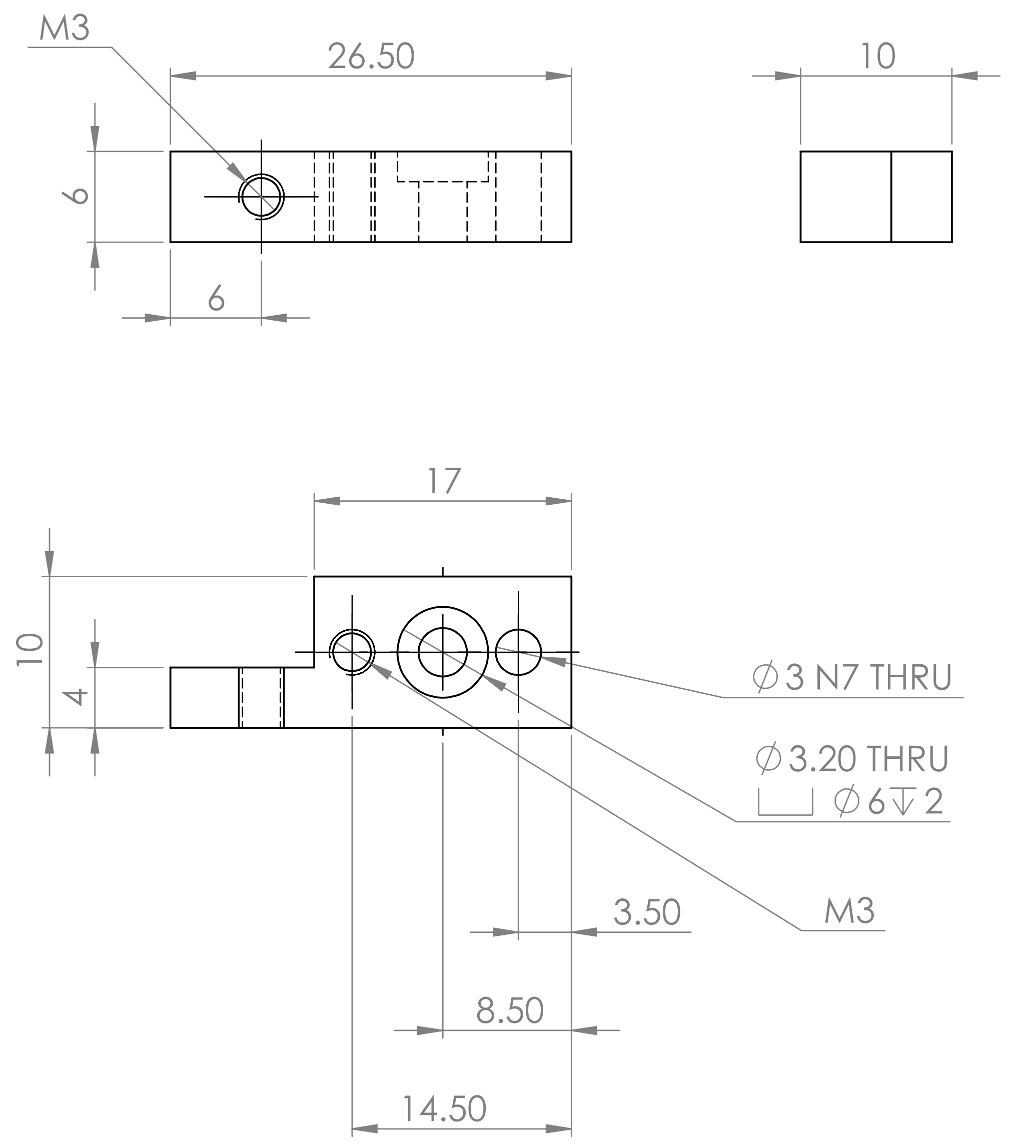

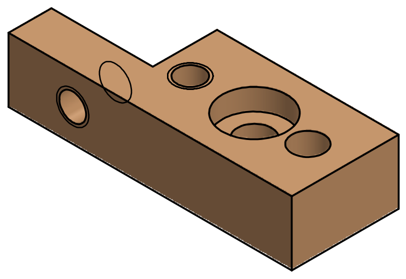

UNLESS OTHERWISE SPECIFIED:<br>DIMENSIONS ARE IN MILLIMETERS<br>SURFACE FINISH:<br>TOLERANCES:<br>LINEAR:<br>ANGULAR:	FINISH:	DEBURR AND BREAK SHARP EDGES	DO NOT SCALE DRAWING	REVISION	

	NAME	SIGNATURE	DATE		
DRAWN					
CHK'D					
APPV'D					
MFG					
Q.A					

TITLE:

Platform_links

MATERIAL: PEI

DWG NO. 190819_block_beda_2

A4

WEIGHT:

SCALE:2:1

SHEET 1 OF 1

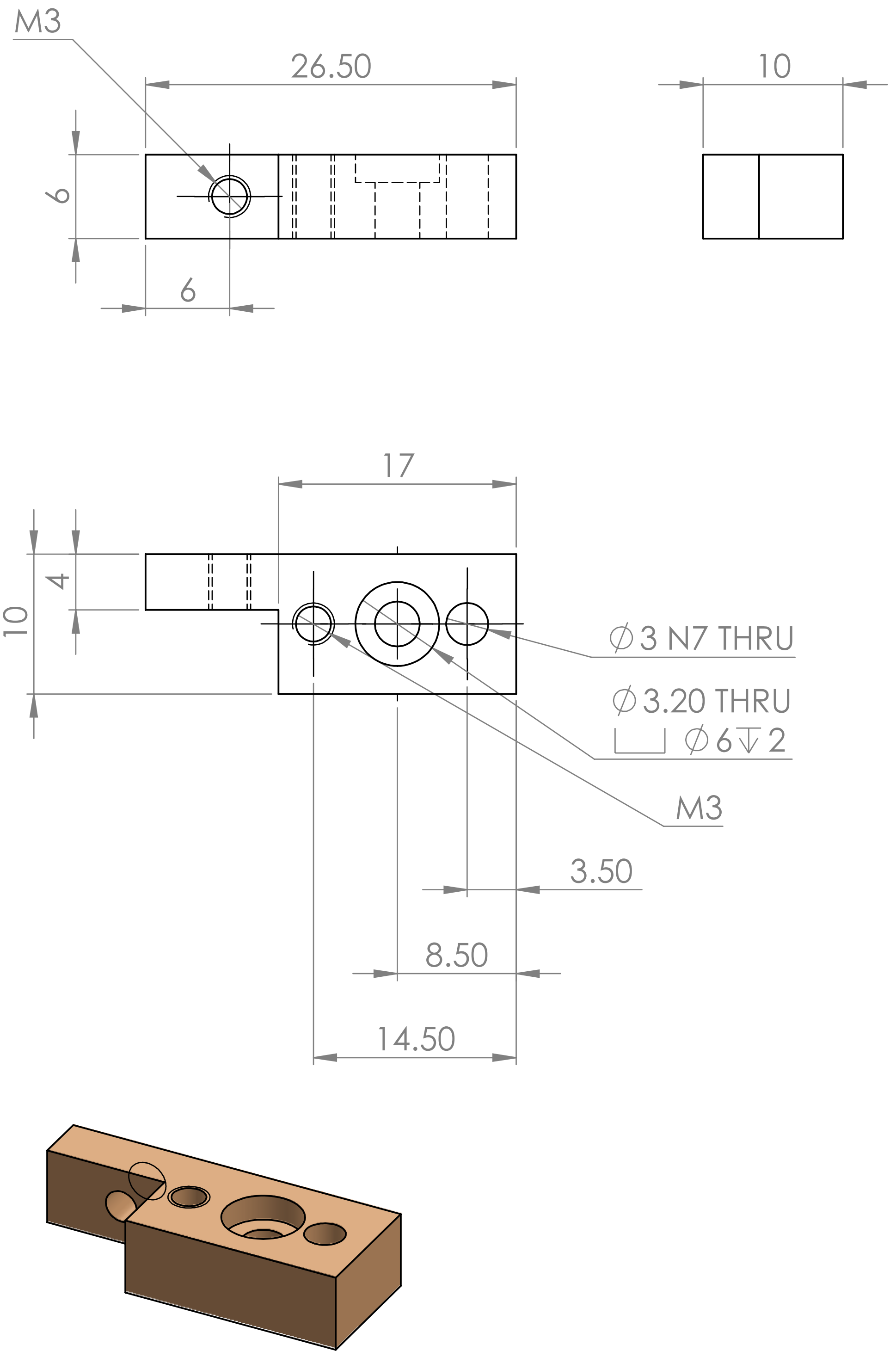

UNLESS OTHERWISE SPECIFIED:
DIMENSIONS ARE IN MILLIMETERS
SURFACE FINISH:
TOLERANCES:
LINEAR:
ANGULAR:

FINISH:

DEBURR AND BREAK SHARP EDGES

DO NOT SCALE DRAWING

REVISION

	NAME	SIGNATURE	DATE
DRAWN			
CHK'D			
APPV'D			
MFG			
Q.A			

TITLE:

Platform_rechts

MATERIAL: PEI

DWG NO. 190819_block_beda_1

A4

WEIGHT:

SCALE:2:1

SHEET 1 OF 1
